# Disentangling charge carrier from photothermal effects in plasmonic metal nanostructures

**DOI:** 10.1038/s41467-019-10771-3

**Published:** 2019-06-17

**Authors:** Chao Zhan, Bo-Wen Liu, Yi-Fan Huang, Shu Hu, Bin Ren, Martin Moskovits, Zhong-Qun Tian

**Affiliations:** 1 State Key Laboratory of Physical Chemistry of Solid Surfaces, College of Chemistry and Chemical Engineering, Collaborative Innovation Center of Chemistry for Energy Materials (iChEM), Xiamen University, 361005 Xiamen, China; 2 Department of Chemistry, University of California, Santa Barbara, CA 93106 USA

**Keywords:** Electrochemistry, Photochemistry, Nanophotonics and plasmonics, Surface patterning, Electrochemistry, Photochemistry, Nanophotonics and plasmonics, Surface patterning

## Abstract

Plasmon-mediated chemical reactions (PMCRs) constitute a vibrant research field, advancing such goals as using sunlight to convert abundant precursors such as CO_2_ and water to useful fuels and chemicals. A key question in this burgeoning field which has not, as yet, been fully resolved, relates to the precise mechanism through which the energy absorbed through plasmonic excitation, ultimately drives such reactions. Among the multiple processes proposed, two have risen to the forefront: plasmon-increased temperature and generation of energetic charge carriers. However, it is still a great challenge to confidently separate these two effects and quantify their relative contribution to chemical reactions. Here, we describe a strategy based on the construction of a plasmonic electrode coupled with photoelectrochemistry, to quantitatively disentangle increased temperature from energetic charge carriers effects. A clear separation of the two effects facilitates the rational design of plasmonic nanostructures for efficient photochemical applications and solar energy utilization.

## Abstract (web summary)

Confidently separating the photothermal effect from the generation of energetic charge carriers and quantifying their relative contribution to chemical reactions remain a great challenge in plasmon-mediated chemical reactions. Here, authors describe a strategy based on the construction of a plasmonic electrode coupled with photoelectrochemistry to quantitatively disentangle these two effects.

## Introduction

Much attention has been paid to the use of nanostructured metals capable of supporting surface plasmons (SPs) to harvest or redistribute incident light for efficient energy conversion, or for carrying out photochemical reactions^1–9^. These processes make use of one or both of the two main consequences of the relaxation of the SPs, namely the production of energetic charge carriers, and the increase in local temperature of the plasmonic system thereby either thermodynamically and/or kinetically increasing the rates and/or yields of chemical reactions^10–14^. Most applications of surface plasmons (SPs), including plasmon-enhanced spectroscopy, sensors, plasmon-mediated chemical reaction (PMCR), and photothermal therapy, are closely related to these two effects^15–18^. For instance, they have been alluded to in accounting for the variation of the intensity of spectroscopic features with time, observed using plasmon-enhanced molecular spectroscopy by proposing that the participating plasmons also induced chemical transformations in the detected molecule during the measurement^19,20^. Importantly, the photo-response of metal nanostructures as a result of the excitation of SPs is quite different from that in semiconductors or dyes because of the absence of a band gap^12,13,20–24^. In plasmonic systems, excited carriers and thermal effects occur simultaneously, making it hard to distinguish them confidently.

Recently, some elegant attempts have been made to address the challenge of distinguishing excited carriers from thermal effects^14,25–28^. One typical strategy is based on temperature control and detection. For example, it is found that the incident light can reduce the thermal activation barrier for ammonia decomposition on a plasmonic photocatalyst^28^. But this strategy requires the accurate measurement of the surface/local temperature of plasmonic catalysts, which is still a great challenge. Another example is using scanning electrochemical microscope (SECM), which has been successfully used to investigate the roles of photoinduced reactions at the substrate and enhanced mass transport to the SECM tip due to the local heating^26^. It is still difficult for SECM to determine whether the chemical reaction on surface is induced by the excited carriers or heating, because SECM can only detect the product diffusing to the probe. Investigating these two effects separately and quantitatively is highly desirable, which enables one to extract the key factors that influence the plasmon-mediated processes. Thereby, the rational design and optimization of plasmonic nanostructures for specific applications can be implemented.

Photoelectrochemistry appears to be one of the most promising tools for tackling such problems, with its high energy and time resolution, and the ease of controlling the Fermi level of the electrodes. In addition, the value of current directly represents the reaction rate. The photoelectrochemical behaviors of semiconductors and dyes have been widely studied and become the basis for their photocatalytic and photovoltaic applications^29–34^. Recently, some key studies have appeared regarding photocurrents in plasmonic metal-semiconductor complexes^35–40^. But the presence of semiconductors complicates the system and prevents a comprehensive understanding of photoelectrochemical behavior of SPs. For example, the contact between the plasmonic nanostructure and semiconductors can greatly influence the charge transfer process^23^. Therefore, it is highly important to study this behavior of pure plasmonic metals first, which not only provides a fundamental understanding of SPs but also will be of significant importance for the various applications of SPs.

Here, we describe the fabrication of a plasmonic photoelectrode with which we probed directly the photoelectrochemical behavior of its constituent plasmonic metal nanostructures and quantitatively unraveled the photothermal from the photoelectronic effect. We find that the plasmonic electrode can function not only as a photocathode, a source of excited electrons, but also as a photoanode, delivering excited holes for an oxidation reaction. The plasmonic photocurrent that we measured following the photoexcitation of this electrode, is found to be readily separable into a rapid response current (RRC) and slow response current (SRC). These, we show, result from the photoelectronic effect and photothermal effect of SPs, respectively; and confirm this conclusion by potential-step and temperature-control studies. These two effects were also systematically studied by separately varying the applied potential, the wavelength of the incident light and its intensity. The results suggest that it is important to properly coordinate these factors in order to optimize the efficiency of the plasmon-mediated chemical (electrochemical) reaction or plasmon-assisted solar energy utilization and to otherwise guide the rational design of the plasmonic structure for a specific application.

## Results

### The plasmonic electrode

**Fig. 1:**
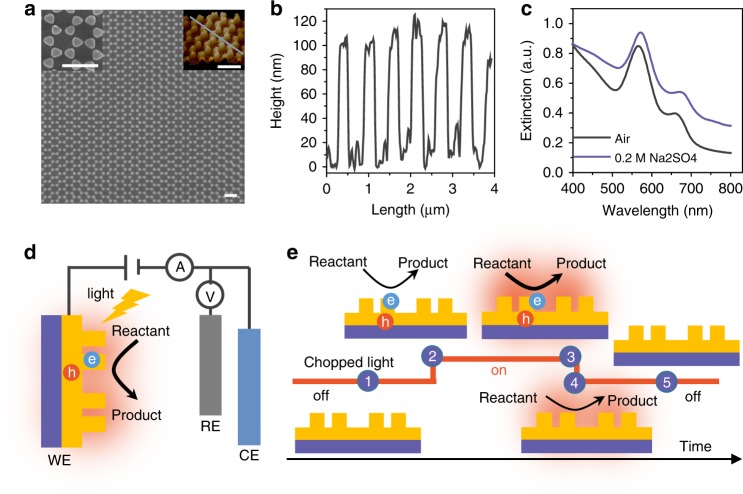
Structures of the as-prepared plasmonic electrode and photoelectrochemical system. **a** SEM and AFM images. **b** The height profile of the as-prepared Au nanoelectrode array along the gray line in AFM image of **a**. **c** The UV-Vis distinction spectrum detected by the specular reflection mode in air and in 0.2 M sodium sulfate solution. **d** Schematic of the three-electrode electrochemical system used in the experiments, including working electrode (WE), counter electrode (CE) and reference electrode (RE). **e** Schematic of photoelectrochemical characterization under the chopped light. The scale bars in **a** are 1 μm. The area of the gold nanoelectrode array is 0.8 cm^2^. The numbers in **e** represent different times

To avoid the influence of surfactant and to achieve good Ohmic contact, we fabricated a plasmonic electrode, a gold nanoelectrode array fabricated using holographic lithography and lift-off process. The plasmonic electrode consisted of a uniform, large-area bowtie array (Fig. 1a) with a period of 600 nm. The detailed fabrication process is described in Supplementary Figs. 1, 2 and Supplementary Table 1. The diameter and height of each nanoelectrode are 250 nm and 100 nm, respectively, as measured by AFM (Fig. 1a, b). The plasmonic electrode shows typical plasmon resonances at ~560 nm and 650 nm, which we attribute to the localized surface plasmon resonance (LSPR) of the bowtie and the surface plasmon polariton (SPP) of the periodic array, respectively (Fig. 1c)^41–43^. Fig. 1d shows the scheme of the plasmonic electrode illuminated with the laser in a three-electrode electrochemical cell. A schematic of the photoelectrochemical process induced by the chopped light is shown in Fig. 1e. On the basis of the accepted mechanism of SPs relaxation^12,13,22^, the energetic charge carriers (electrons and holes) generated upon illumination decay in several ps, which can directly impact the chemical reactions. As has been well observed and investigated in semiconductor electrodes or plasmonic metal-semiconductor complexes^12,13,39,40^, the photocurrent induced by energetic charge carriers responds immediately upon illumination, and reaches maximum usually in less than several ms (2 in Fig. 1e). Whereas, it usually takes a longer time (seconds) to reach the thermal diffusion equilibrium for the heat-induced temperature increase of the surroundings (3 in Fig. 1e). Accordingly, a photoelectrochemical method with a millisecond time resolution would be able to disentangle the charge carrier effect from photothermal effect. Specifically, turning the light off would cause the photoelectronic effect to disappear rapidly, while the increased temperature declines more slowly (4 in Fig. 1e).

### Photoelectrochemical studies

**Fig. 2:**
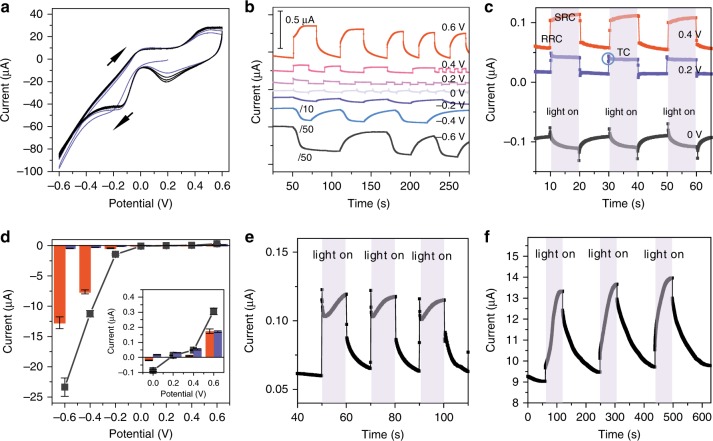
The photo-electrochemical properties of the plasmonic electrode. **a** The typical CV curve of Au nanoelectrode array. **b** The photocurrent of the Au nanoelectrode array at different applied potentials, and the current-time curves at negative potentials have been divided by 10 for −0.2 V and 50 for −0.4 and −0.6 V. **c** The current-time curves of the Au nanoelectrode array at 0.4 V, 0.2 V, and 0 V. The current changed immediately when illuminating the electrode, and the plasmonic photocurrent could be divided in two parts according to the response time: the RRC (0.05 s) and the SRC (10 s). The transient current (TC) is marked by blue cycle. **d** The background current, RRC (blue) and SRC (red) as a function of applied potential. The background current is the dark current without the illumination. The RRC corresponds to the current change in the first 0.05 s. The SRC corresponds to the current change in the next 10 s for the case showing transient current, the SRC was calculated after the transient response. **e** Typical photocurrent of Au nanoelectrode array at 0.6 V in deaerated 0.2 M sodium sulfate electrolyte. **f** Typical photocurrent of Au nanoelectrode array at 0.4 V in deaerated 0.25 mM ferrocenemethanol solution. The error bars represent the standard deviation

Using the Au nanoelectrode array as the working electrode, we carefully investigated the photoelectrochemical behavior of SPs when the nanoelectrode array was illuminated with light of wavelength larger than 420 nm, and power density of 300 mW cm^−2^, which is of the order of the power density of sunlight (AM 1.5, 100 mW cm^−2^). The cell consists of three electrodes: a gold nanoelectrode array, a Pt foil counter electrode and a saturated calomel reference electrode. The electrolyte is 0.2 M sodium sulfate. A cyclic voltammogram of the Au nanoelectrode array shown in Fig. 2a displays the electrochemical behavior typical for a pure gold electrode in a neutral electrolyte (Supplementary Fig. 3)^44^. However, the photocurrent produced by the Au nanoelectrode array is ~40-fold greater (Fig. 2b) than what a smooth Au film produces (Supplementary Fig. 4). Its photoelectrochemical characteristics differ markedly from those of semiconductors. Specifically, we observed two interesting phenomena.

First, upon illumination, the current was enhanced relative to the open circuit potential (OCP, which occurs at ~0.2 V, Supplementary Fig. 5) when using both negative potential (producing a cathodic photocurrent, ~13 μA at −0.6 V) and positive potential (producing an anodic photocurrent, ~0.4 μA at 0.6 V). In other words, the incident light can promote both oxidation reactions (e.g., reversible gold oxidation) and reduction reactions (e.g., oxygen reduction or hydrogen evolution). This contrasts with what is observed with semiconductor-based photoelectrochemistry, where an n-type or p-type semiconductor normally functions as either an oxidation or reduction photoelectrode but not both;^31–33^ for example, an n-type semiconductor such as TiO_2_ usually functions as a photoanode^32,33^. In contrast, on a plasmonic electrode, the transfer directions of the excited electrons or holes can be reversed by applying an appropriate potential. For example, at −0.6 V, excited electrons from the electrode are transferred to species in solution to promote hydrogen evolution^10,45–47^. For plasmonic metal-semiconductor electrode, the direction and efficiency of the charge transfer process are highly related to the band structure of the semiconductor and the Fermi level of the plasmonic nanostructure. Moreover, the interface between them can also greatly influence the charge transfer direction and efficiency, which is yet to be more extensively investigated and sophisticatedly controlled.

Second, the photocurrent curve is composed of two regions: a rapid response current (RRC) region operating on a time scale of 0.05 s and a slow response current (SRC) region, on a time scale of 10 s (Fig. 2c, Supplementary Figs. 6, 7). In addition, a transient current at the time scale of less than 0.5 s, (marked as blue cycle) appears immediately after the RRC process when the SRC was suppressed at 0.2 V, which resembles the transient currents observed in semiconductor systems^48^. The positive charge passed during this transient current was measured to be ~5 nC, which is too small to be a charge associated with a reaction involving a monolayer of molecules at the surface. We, therefore, attribute the transient to a capacitive current which involves charges trapped in surface states and/or the charges required to charge the capacitive electric double layer induced by the incident light^49,50^. Similar photocurrent response to RRC has been observed and investigated in semiconductor electrodes and plasmonic metal-semiconductor complexe^29–34^, and has been attributed to excited carriers. Therefore, we assign the RRC observed in the plasmonic electrode to excited carriers^35–40^.

Figure 2d shows a plot of the RRC and the SRC as a function of the applied potential. Both increase with the increased potential relative to the OCP. It should be emphasized that the plasmonic photocurrent at the negative potential is much larger than that at the positive potential (Fig. 2b). Different from the opposite polarity of RRC to the background current at 0 V, the SRC follows the same polarity and trend as the background current (the dark current). The background current is mainly the Faradaic current due to the redox reaction on the electrode, indicating the SRC is related to the electrochemical reaction (Supplementary Figs. 8, 9). For example, at 0.2 V (near the OCP), although the RRC is very obvious, the SRC is almost zero (Fig. 2c). If so, one expects to see a change in the SRC if the electrochemical reaction is varied; and indeed, removing oxygen from solution by de-aerating (with nitrogen) sharply reduces the SRC in response to the decreased oxygen concentration (Fig. 2e, S10). However, adding ferrocene to the solution while keeping the electrode potential at the oxidation potential, caused the SRC to increase (Fig. 2f, Supplementary Fig. 11). In addition, the temperature increase (~0.03 K, Supplementary Fig. 12) in the solution is negligible under our experimental condition. The long response time, and its strong dependence on the electrochemical reaction strongly suggests that the SRC is associated with photothermal processes of SPs.

### Potential-step and temperature-control experiments

**Fig. 3:**
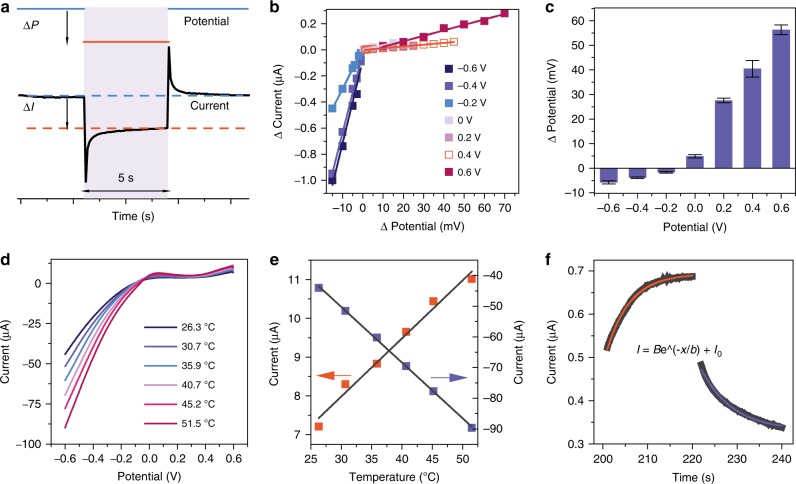
The potential-step and temperature-control experiments. **a** The typical *i*−*t* curve in response to a potential step, Δ*I* is the current change induced by the step potential (Δ*P*) after a 5 s interval to stabilize. **b** The change of current as a function of the step potential at various applied potentials. **c** Step potential required to achieve a similar current change as was achieved with the RRC at various applied potentials. **d** The *I*–*V* curve of the Au nanoelectrode array at various temperature. **e** The current at −0.6 V (blue) or 0.6 V (red) as the function of the temperature. **f** The fitting of the SRC at 0.6 V, the black dots are the experimental result (0.6 V 200–240 s); the red lines are the fitted functions under illumination; the blue line are the fitted functions in the dark. The error bars represent the standard deviation

To further investigate the validity of our attributions of the SRC and RRC, we performed potential-step and temperature-control experiments in the dark (details were shown in the section of electrochemical and photoelectrochemical measurements in Supplementary Methods). It has been discovered that SPs can generate the electric potential under the light illumination, namely the plasmoelectric potential, which converts the optical energy into the electrical energy^51^. Our experiments show that the illumination of the Au nanoelectrode array can enhance the electrochemical reaction and lead to a RRC similar to the potential-step (*i*−*t*) curve. The similarity suggests that we can use the potential step method to mimic the RRC process. Accordingly we carried out a series of experiments at different potential in which the step potential (Δ*P*) was adjusted to achieve a current change (Δ*I*) similar to that obtained using light during the RRC process (Fig. 3a). This approach is equivalent to regarding Δ*P* as the plasmoelectric potential induced by the incident light in the electrochemical system. Doing so, allows us to simulate the electrochemical responses we obtained via illumination at different applied potentials by applying the equivalent plasmoelectric potentials. Figure 3b shows a plot of Δ*I* as a function of Δ*P*. For most potential values the current response stabilizes within 5 s after the potential step, and Δ*I* varies linearly with potential. Figure 3c shows the Δ*P* values required to achieve comparable photocurrents (RRC) corresponding to the indicated applied potentials, which would also correspond to the reported plasmoelectric potential (at OCP). (The *i*–*t* curve at various potentials simulating the photoelectronic process is shown in Supplementary Fig. 13.) More interestingly, these Δ*E* values increase with increasing applied potential in the positive or negative regions, which suggests that the applied potential can promote the plasmoelectric potential, reinforcing the speculations outlined above regarding the RRC.

Figure 3d shows the voltammograms of the Au nanoelectrode array at different temperatures (*T*). The currents at both 0.6 V (red square) and −0.6 V (blue square) show a linear dependence on the temperature (Fig. 3e), which were fitted to Eq. 1:

1 $$I = A_0T + D$$

*I* is the current, *A*_0_ and *D* are constants. If the SRC is a result of the photothermal effect induced temperature increase associated with SPs, it should follow the temperature-time curve. The system is an open system being heated at a constant light intensity. The thermal dynamics on the basis of the linear non-equilibrium thermodynamics yields the following expression:

2 $$T = Ae^{ - \frac{{ak}}{{Cl}}t} + \frac{{Pl}}{{ak}} + T_0$$

*P* is the energy input by the incident light, *a* is the size of the electrode, *l* is the thickness of the thermal diffusion layer in which the temperature changes linearly, *t* is the time, *T*_*0*_ is the external temperature considered to be constant, *k* is the thermal conductivity, *C* is the heat capacity of the system, and *A* is a constant determined from boundary conditions. Assuming *T* = *T*_0_ at *t* *=* 0, we obtain Eq. 3:

3 $$A = - \frac{{Pl}}{{ak}}$$

in which, *A* must be negative because the energy input by the incident light is positive. In contrast, for the SRC recorded in the dark state, *A* is positive (details were shown in Supplementary Table 2). The excellent fit of the experimental SRC to the derived equations, shown in Fig. 3f, strongly supports our conclusion that the SRC is induced by the photothermal effect following plasmon decay.

### Dependence of plasmonic photocurrent on incident light

**Fig. 4:**
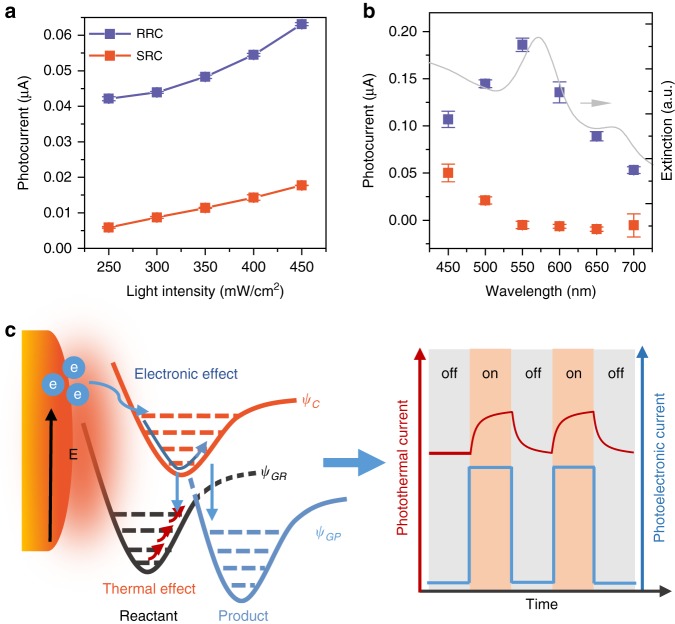
The photoelectronic current and photothermal current. **a** The RRC and SRC as a function of the incident light intensity. **b** The RRC and SRC as a function of the incident light wavelength. **c** The mechanism for the photoelectronic effect and photothermal effect of SPs on the electrochemical reaction. The schematic potential energy surface was simplified by using Reactant and Product. *ψ*_*GR*_ represents the ground state of the reactant; *ψ*_*C*_ represents the charged state of the reactant; and *ψ*_*GP*_ represents the ground state of the product. The error bars represent the standard deviation

Such photoelectrochemical methods allow us to distinguish between the plasmon-mediated photoelectronic and photothermal effects to quantitatively investigate contributions of these two effects to chemical reactions. The dependences of the plasmonic photocurrents on the light intensity and wavelength at 0.6 V free of oxygen are shown in Fig. 4a, b (Supplementary Fig. 14). The RRC shows a super-linear dependence on the incident light intensity, which was fitted by polynomial (Supplementary Fig. 15), in contrast to the linear dependence of the SRC. Such a response has been suggested as a hallmark of the hot-carrier-driven chemistry of the plasmonic nanostructure^5,18,52^. Fig. 4b shows the wavelength dependence of the photocurrent, assisted by using bandpass filters to obtain the desired wavelength (Supplementary Fig. 16). The RRC is in good agreement with the UV-Vis extinction spectrum (LSPR part) of the Au nanoelectrode array, which indicates that the photoelectronic effect depends on the plasmonic properties of the device. We did see a small deviation of RRC curve to the extinction spectrum, which is understandable, as the PMCR system is a complex reaction system including surface plasmons, excited carriers, local heating and chemical reactions^7,37,46^. Two more facts may further contribute to the deviation: one is the energy distribution of the excited carriers generated under different wavelength which can influence the probability to induce chemical reactions and the other is the effects of SPP on the chemical reaction. In contrast, the SRC increases with the decreasing wavelength and becomes obvious at the incident light wavelength shorter than 550 nm (corresponding to the interband transition from d to s band), which indicates the interband transition makes the major contribution to the photothermal current. Whereas, under the illumination with light of a longer wavelength (at around LSPR wavelength), the energetic excited carriers of SPs participate in the chemical reaction, they will not further thermalize (Fig. 4b). This is a unique feature of excited carriers and photothermal effect in the PMCR system. For a Au film without SPs, we could only detect small photocurrents when the illumination wavelength is shorter than 450 nm (Supplementary Fig. 17).

For PMCR, the excited carriers can directly transfer to reactants to form a charged species to greatly enhance the reaction rates, or use their excess energy to overcome the energy barriers required for the reaction^1,5,9,10,13^. Alternatively, the photothermal effect may increase the population of activated molecules with a high kinetic energy, rotational energy, and even vibrational energy to promote the chemical reaction. The experimental photocurrent curve demonstrates that both the photothermal and photoelectronic effects of SPs can influence the chemical reaction and response on different time scales, which can be quantitatively disentangled by the photoelectrochemical method (Fig. 4c). Distinguishing the contribution of the photoelectronic effect from the photothermal effect and extracting the key factors that influence the PMCR, we can be better positioned to rationally design plasmonic structures with appropriately controlled ratios of these two contributing effects to gain the optimal efficiency for specific chemical reactions.

## Discussion

In summary, we have demonstrated the photoelectrochemical response of a plasmonic Au nanoelectrode array, whose properties differ dramatically from those of semiconductors or dyes. We find that the plasmonic photocurrents can be enhanced at both negative and positive potentials, and that the direction of the photocurrent can be varied by a proper choice of the applied potential, thereby using the plasmonic electrode to promote either reduction reactions by excited electrons or oxidation reactions by excited holes. The plasmonic photocurrent consists of two components: a rapid response current (0.05 s) and a slow response current (10 s), corresponding, respectively, to photoelectronic (excited carriers) and photothermal (local heating) effects. The wavelength dependence of the photoelectronic current tracks the SPR absorption spectrum, and shows a super-linear dependence on the incident light intensity, while the photothermal current is only linear over a certain light intensity range, and becomes obvious in the interband transition region. Using the above-described photoelectrochemical, we were able to disentangle the coupled photoelectronic and photothermal effects quantitatively, potentially guiding one’s ability to optimize the design of plasmonic nanomaterials for specific applications, including plasmon-mediated chemical reactions and photothermal therapy, by synergistically coupling those two effects.

## Methods

### Fabrication of electrode

The plasmonic electrodes were fabricated using two main techniques: holographic lithography (HL) and lift-off. The glass substrate slides were cleaned by sonication in acetone and ethanol for 10 min, followed by soaking in piranha solution (H_2_SO_4_/H_2_O_2_ = 3:1, volume ratio) for 30 min and rinsing with deionized water. They were dried with the high purity nitrogen gas. A 100 nm Au layer was deposited on the cleaned glass slides using electron-beam evaporation (Temescal, FC-2000). Lift off resist (100 nm) and negative photoresist SU-8 (500 nm) layers were successively spin-coated on the pre-deposited gold film (100 nm) at, respectively, 5000 rad/min and 4000 rad/min. Holographic lithography was carried out using 266 nm laser light and developed using propylene glycol methyl ether acetate forming the SU-8 template, as shown in Fig. S1b. Reactive ion etching (RIE) was used to remove the exposed lift-off resist from the SU-8 template, and another gold layer with a thickness of 100 nm was deposited on the template. The sample was dipped in acetone solution to dissolve away the remaining resist thereby forming the Au electrode array. Finally, the as-prepared gold array structures were cleaned with piranha solution and rinsed with deionized water then fixed the areas by insulating cement.

### Characterizations of the electrode

Scanning electron microscopy (SEM, S4800) was employed to image the as-prepared electrodes. Energy dispersive X-ray (EDX, S4800) was used to detect the surface composition. AFM (Bruker) was used to confirm the morphology details of as-prepared electrodes. The UV-Vis absorption spectrum was acquired by the Carry 5000 with the specular reflection model (Agilent).

### Electrochemical and photo-electrochemical measurements

All the electrochemical and photoelectrochemical experiments were performed in a three-electrode setup with the Au nanoelectrode array as the working electrode, a Pt plate as counter electrode and saturated calomel electrode as the reference electrode, 0.2 M sodium sulfate aqueous solution was used as the electrolyte. Cyclic voltammograms were recorded at a scan rate of 0.05 V/s and a potential interval of 0.001 V (sample frequency 0.02 s). The potential-step experiment was carried out by chronoamperometry using appropriate step potentials for the various potentials, a pulse width of the step potential of 5 s, and a sampling interval of 0.05 s. A 300 W Xe lamp with an optical filter (λ > 420) (unless otherwise stated) was used as light source in the photo-electrochemical experiments. The light intensity at the electrode surface was 300 mW/cm^2^. Inactive areas of the electrodes were covered by an insulating and opaque cement. Photocurrents were measured by chronoamperometry with a sampling interval of 0.02 s. The electrochemical experiments without the oxygen were carried out by saturating with N_2_ gas for 30 min. The wavelength dependence experiments were carried out by filtering the light source output using appropriate band-pass filters, the light intensity at the electrode surface is 40 mW/cm^2^.

## Supplementary information

Supplementary Information

Peer Review

## Data Availability

The data that support the findings in this study are available from the corresponding author upon request.
