# Supplementary material for: Disentangling charge carrier from photothermal effects in plasmonic metal nanostructures
Source: Nat Commun. 2019 Jun 17;10:2671. doi: 10.1038/s41467-019-10771-3 (PMC6572789; doi:10.1038/s41467-019-10771-3)
Supplement: Supplementary file 1 — Supplementary Information [file 41467_2019_10771_MOESM1_ESM.pdf]

Supplementary information

Disentangling charge carrier from photothermal effects in plasmonic metal nanostructures

Zhan et al.

Supplementary Methods

Chemicals: The Na_2SO_4 and ferrocenemethanol were purchased from Sigma. Acetone, ethanol, sulfuric acid (98%) and hydrogen peroxide (30%) were purchased from Sinopharm Chemical Reagent Co., Ltd. The microscope slides (Sail Brand, China) with the size of 25.4 x 25.5 x1 mm were used as the substrates here. The SU-8 2000.5 photoresist and lift-off resist (LOR) were purchased from MicroChem, Germany. All materials were directly used in our experiment without any further purification.

Fabrication of electrode: The pure plasmonic electrodes with a high uniformity over a large area were fabricated by two main steps: holographic lithography (HL) and lift-off, as shown in Fig. S1. Glass slides as the substrate were carefully cleaned by sonicating in acetone and ethanol for 10 min, followed by soaking in piranha solution (H_2SO_4/H_2O_2 = 3:1, volume ratio) for 30 min and rinsing with deionized water. Then the slides were dried with the high purity nitrogen gas. The 100 nm Au layer was deposited on the cleaned glass slides using electron-beam evaporation (Temescal, FC-2000). After that, the lift off resist (100 nm) and negative photoresist SU-8 (500 nm) layers were successively spin coated on the top of the pre-deposited gold film (100 nm) with the rotating speed of 5000 rad/min and 4000 rad/min. The prepared substrates were then exposed to a 266 nm laser using the HL method, followed by development with propylene glycol methyl ether acetate. As shown in Fig. S1b, the patterned SU-8 template was formed after development. The reactive ion etching (RIE) was applied to remove the exposed LOR layer on the bottom of the SU-8 template. After that, another gold layer with a thickness of 100 nm was deposited on the template. Dipping the whole sample into the acetone solution, as a result of the dissolution of the LOR layer, the Au electrode array was formed. Finally, the as-prepared gold array structures were cleaned with piranha solution and rinsed with deionized water then fixed the areas by insulating cement.

Characterizations of the electrode: Scanning electron microscopy (SEM, S4800) was employed to observe the size and morphology of as-prepared nanoelectrode arrays. Energy dispersive X-ray (EDX, S4800) was used to detect the surface composition. AFM (Bruker) was used to confirm the morphology details of as-prepared electrodes. The UV-Vis absorption spectrum was acquired by the Carry 5000 with the specular reflection model (Agilent). The reflection model is chosen because that our periodic substrate has a strong directional reflection, which does not meet the basic requirement of diffuse reflectance measurement.

Electrochemical and photoelectrochemical measurements: All the electrochemical and photoelectrochemical experiments were performed in a three-electrode setup with the Au nanoelectrode array as the working electrode, a Pt plate as the counter electrode and saturated calomel electrode as the reference electrode. 0.2 M sodium sulfate aqueous solution was used as the electrolyte. The cyclic voltammogram was measured with a scan rate of 0.05 V/s and sample interval of 0.001 V (sample frequency 0.02 s). The potential-step experiment was carried out by the chronoamperometry technique with the required step potential at different potentials. The pulse width of the step potential is 5 s and the sample interval is 0.05 s. For photoelectrochemical experiments, the light source was a 300 W Xe lamp with an optical filter ($\lambda > 420$) unless otherwise stated and the light intensity at the electrode surface was 300 mW/cm^2. Prior to measurement, the inactive areas of the electrodes were covered by the insulating cement. The photocurrent was measured by chronoamperometry technique at some typical potentials with the sample interval of 0.02 s. The electrochemical experiments free of oxygen were carried out by saturating the solution with N_2 gas for 30 min. The wavelength dependence experiments were carried out by using the band-pass filters, which only allow photons with certain wavelength to pass, the light intensity at the electrode surface is 40 mW/cm^2.

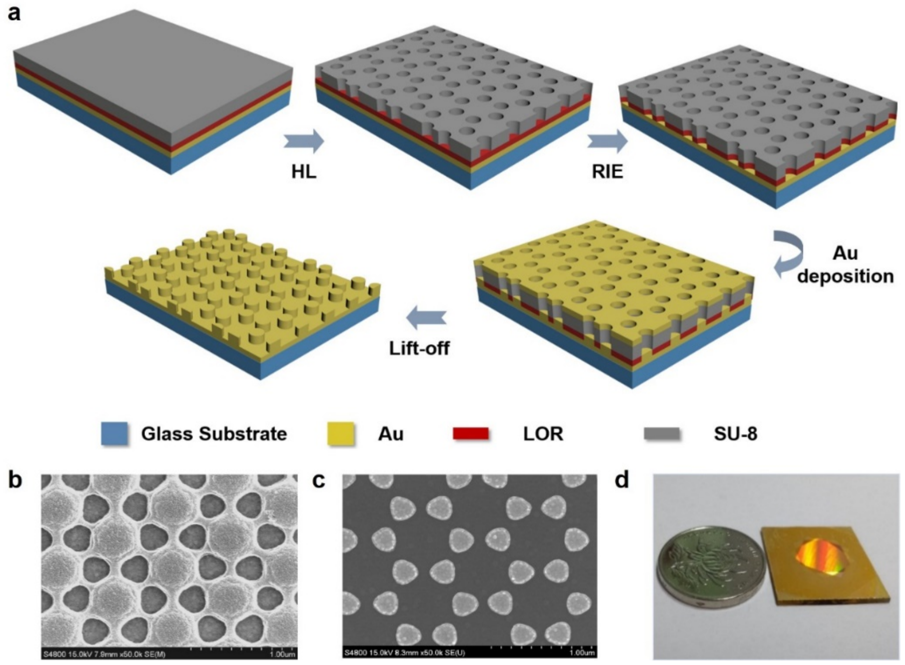

Supplementary Figure 1. **The fabrication of the pure plasmonic electrode.** (a) The schematic of

fabrication process of the gold nanoelectrode array. (b) The SEM image of photoresist template. (c) The final gold nanoelectrode array. (d) A picture of the plasmonic substrate in comparison with a one-yuan coin.

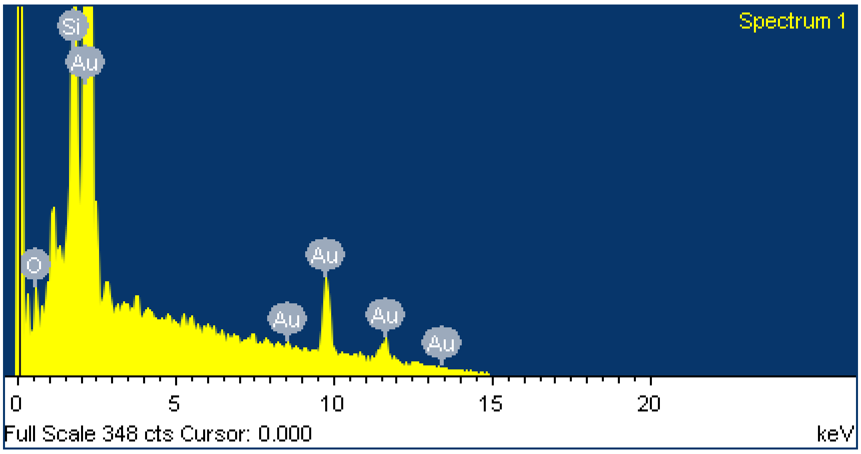

Supplementary Figure 2. The EDS image of as-prepared plasmonic electrode, demonstrating the cleanliness of the surface.

Supplementary Table 1. The weight and atomic ratio of elements from as-prepared plasmonic electrode from EDX.

Element	Weight%	Atomic%
O K	2.40	19.41
Si K	4.13	19.08
Au M	93.47	61.51
Totals	100.00	100.00

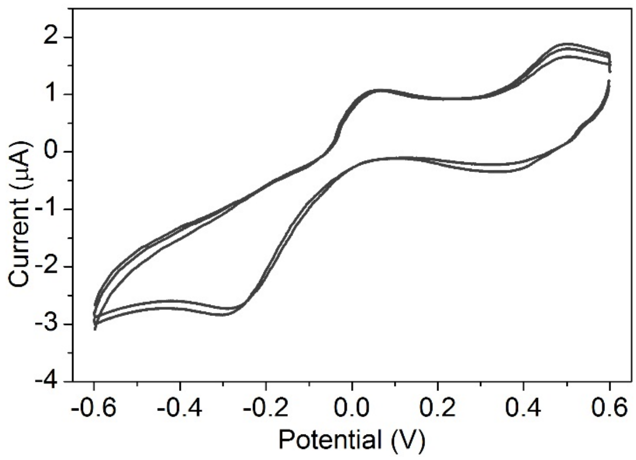

Supplementary Figure 3. The CV curve of a flat Au electrode without nanostructures. It shows a similar electrochemical behavior to that of a Au nanoelectrode array.

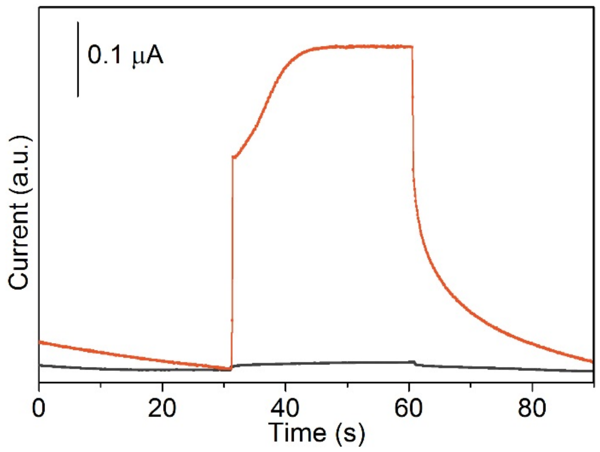

Supplementary Figure 4. The photocurrents of Au nanoelectrode array and Au film with a same area (0.8 cm^2) under the visible light at 0.6 V. For the Au nanoelectrode array with SP, the total photocurrent is 0.43 μA and the photocurrent of Au film electrode without SP is only 0.01 μA.

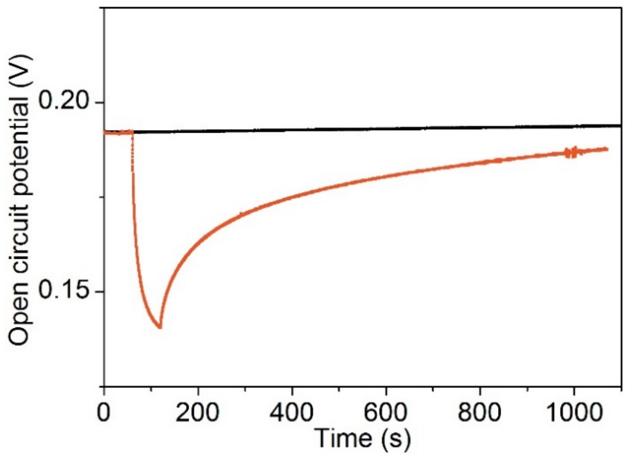

Supplementary Figure 5. The response of the open circuit potential (OCP) of the Au nanoelectrode array while switch on and off the visible light (red). The black curve is the OCP without the incident light. The OCP was changed about 50 mV after the 120 s illumination.

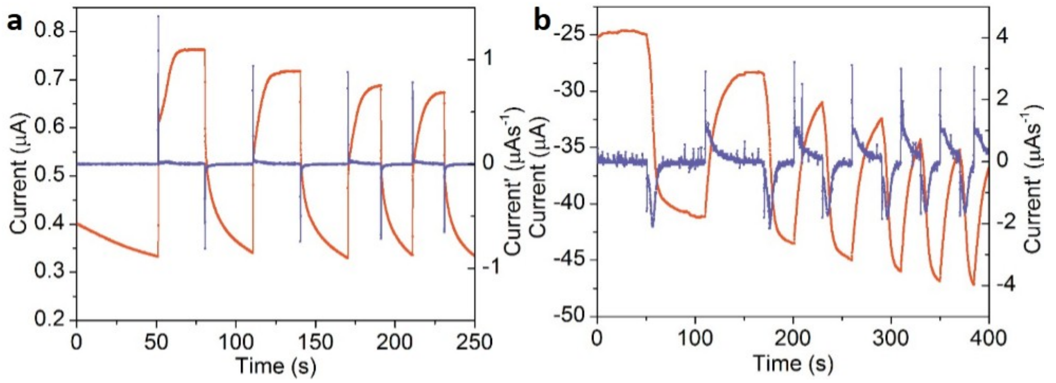

Supplementary Figure 6. The photocurrent and their differential at two typical applied potentials, 0.6 V (a) and -0.6 V (b). Both of them could be separated into two components, the rapid response current (RRC) and the slow response current (SRC), even with a large background current at the negative potential.

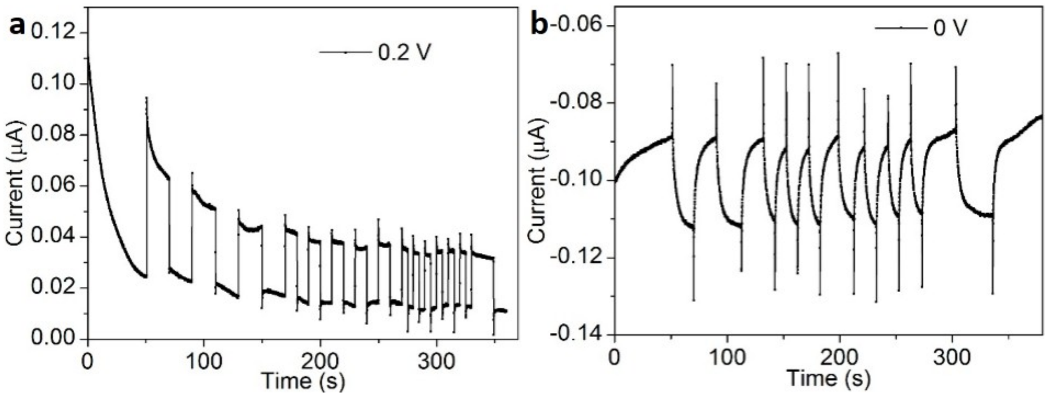

Supplementary Figure 7. The photocurrent of Au nanoelectrode array at 0.2 V (a) and 0 V (b). At 0.2 V (near the open circuit potential), the SRC is nearly unobservable, and the photocurrent response is similar to that in the potential step experiment. At 0 V, the directions of the RRC and SRC are different which means the effects inducing the RRC and SRC are different and their influences on the current are different.

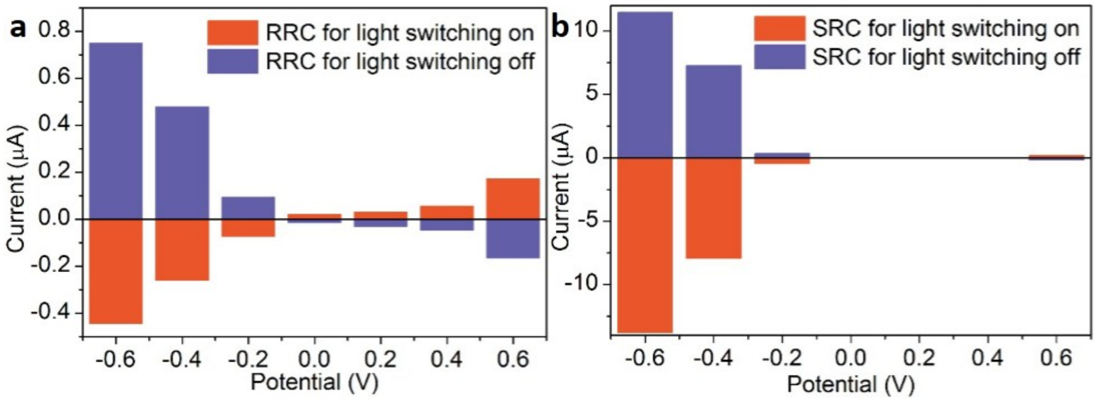

Supplementary Figure 8. The rapid response current (a) and the slow response current (b) response to the light switching. All of the applied potentials (negative or positive) can significantly improve the rapid response currents, such as -0.6 V or 0.6 V, which is similar to the photo-anode at positive potential and photo-cathode at negative potential. But for the SRC, it matches well with the background current.

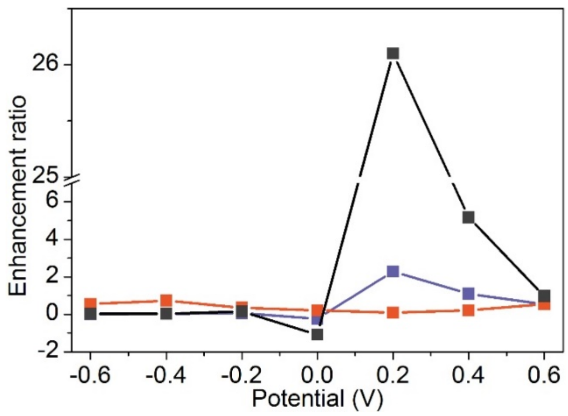

Supplementary Figure 9. The enhancement ratio of the incident light, calculated by dividing the SRC (the red one) and RRC (the blue one) by the background current or dividing the RRC by the SRC (the black one).

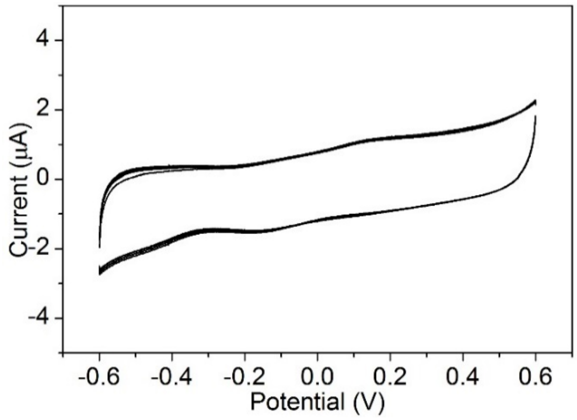

Supplementary Figure 10. The CV curve of Au nanoelectrode array by saturating the solution with N_2 gas for 30 min. The oxygen reduction at -0.2 V cannot be observed.

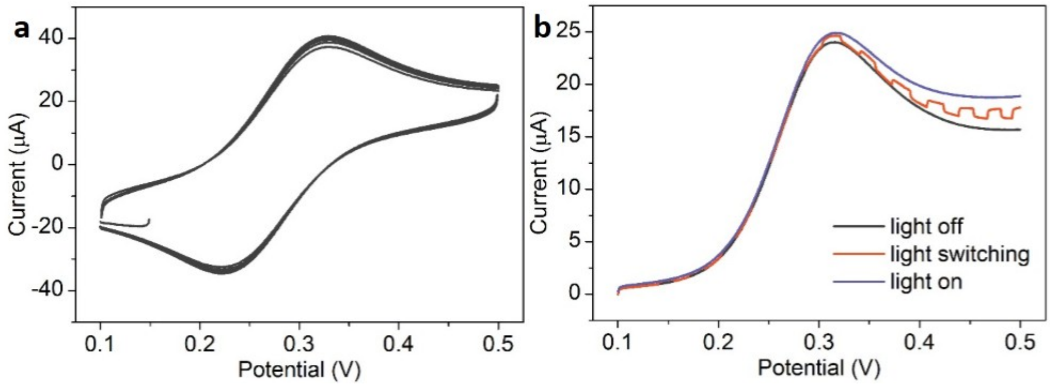

Supplementary Figure 11. (a) The CV curve of a Au nanoelectrode array in 0.25 mM ferrocenemethanol aqueous solution with 0.2 M Na_2SO_4 under the dark condition, (b) the linear voltammogram of a Au nanoelectrode array in 0.25 mM ferrocenemethanol aqueous solution with 0.2 M Na_2SO_4 under different conditions.

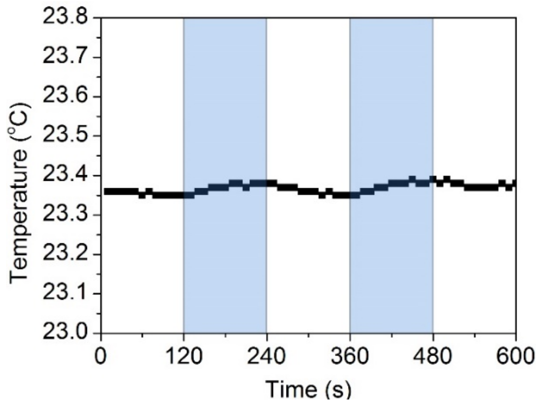

Supplementary Figure 12. The temperature changing of the medium with the chopped incident light. The temperature increase is about 0.03 K under the experimental condition, which is negligible.

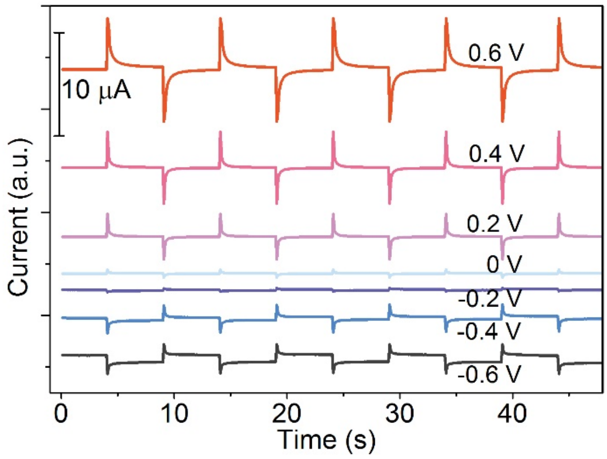

Supplementary Figure 13. Typical potential step curves at different applied potentials which have comparable current change compared with the RRC at the corresponding potentials. The corresponding step potential is shown in Figure 3c.

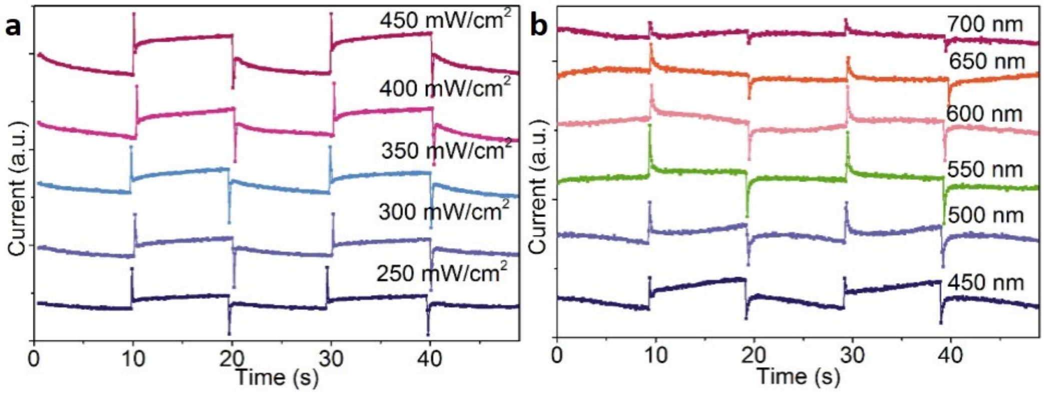

Supplementary Figure 14. (a) The photocurrent of a Au nanoelectrode array at different light intensities free of oxygen, (b) the photocurrent of a Au nanoelectrode array illuminated with light of different wavelengths free of oxygen.

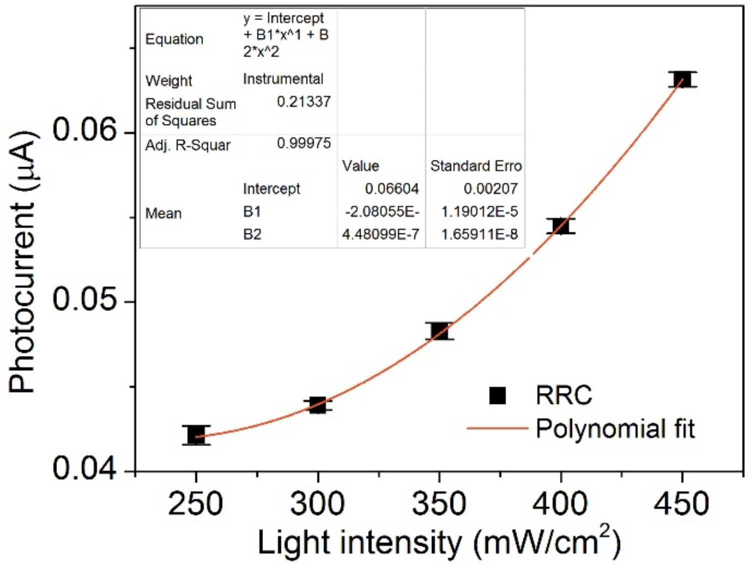

Supplementary Figure 15. The RRC at 0.6 V as a function of the incident light intensity and the fitting result as indicated in the figure.

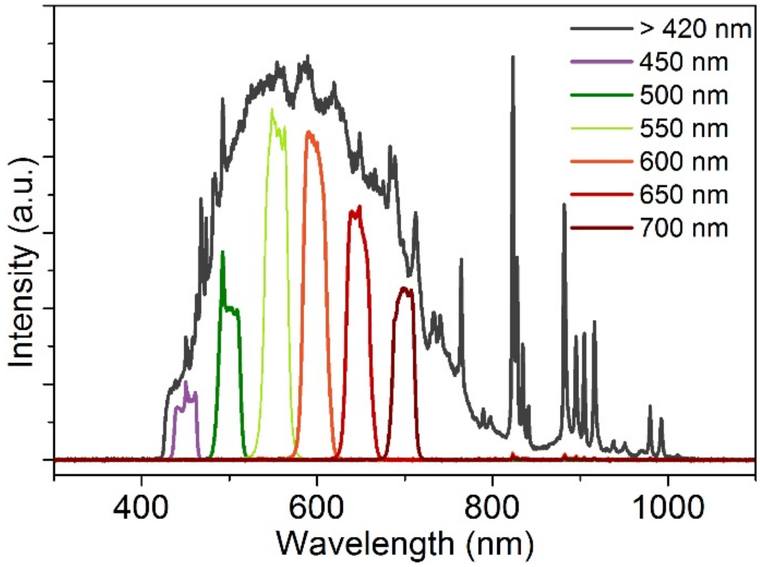

Supplementary Figure 16. Spectrum of the light source with 420 nm long-pass filter, and 450 nm, 500 nm, 550 nm, 600 nm, 650 nm, 700 nm band-pass filters.

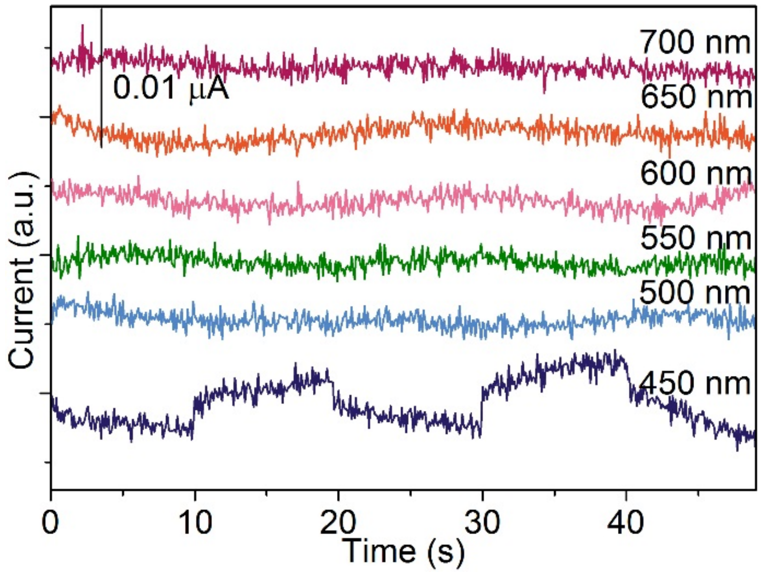

Supplementary Figure 17. The photocurrent of a Au film electrode illuminated with light of different wavelengths free of oxygen. Only under 450 nm illumination (interband transition region), the photocurrent becomes obvious.

Supplementary Table 2. The fitting results of the SRC with the equation "I = A×exp(-x/b) + I_0". The former one is the SRC with the light on; the other one is the SRC with the light off.

Reduced Chi-Sqr	$4.03*10^{-6}$
Adj. R-Square	0.998
Constant	Value
I_0	0.694
A	$-1.24\times10^{+16}$
b	5.18
Reduced Chi-Sqr	6.22×10^{-6}
Adj. R-Square	0.995
Constant	Value
I_0	0.330
A	$3.82\times10^{+12}$
b	7.18

Supplementary Discussion

Derivation process for the thermal dynamics in our system

The system is an open system being heated at constant power by illumination. *P* is the energy input by the incident light, *a* is the size of the electrode, *l* is the thickness of the thermal diffusion

layer in which the temperature changes linearly, t is the time, T_0 is the external temperature, considered to be constant, k is the thermal conductivity, C is the heat capacity of the system. Thus we can obtain the following equation:

$$P = \frac{dT}{dt}C + ak\frac{T - T_0}{l}$$

Integrating it over time, we can get the following equation:

$$T = Ae^{-\frac{ak}{Cl}t} + \frac{Pl}{ak} + T_0$$

A is constants that is determined from boundary conditions. Assuming $T = T_0$ at $t = 0$, we obtain equation:

$$A = -\frac{Pl}{ak}$$

where, A is negative because the energy input by the incident light is positive.
