# Supplementary material for: Disentangling charge carrier from photothermal effects in plasmonic metal nanostructures
Source: Nat Commun. 2019 Jun 17;10:2671. doi: 10.1038/s41467-019-10771-3 (PMC6572789; doi:10.1038/s41467-019-10771-3)
Supplement: Supplementary file 2 — Peer Review [file 41467_2019_10771_MOESM2_ESM.pdf]

Editorial Note: Parts of this peer review file have been redacted as indicated to remove third-party material where no permission to publish could be obtained.

Reviewers' comments:

Reviewer #1 (Remarks to the Author):

Zhan et al. describe a photoelectochemical method for separating plasmon-enhanced photocurrent into its constituent contributions from i) photothermally induced temperature rise and ii) photoinduced charge separation. The photoelectrochemical characterization is systematic - the effects of temperature and applied potential on the photocurrent are studied -- but the work is ideally suited for an analytical electrochemistry audience, which would be able to appreciate the merits of the technique used by the authors. From the standpoint of new insights or knowledge or broad appeal, the paper is rather limited.

1) First, it is well appreciated in the plasmonic catalysis and plasmonic electrochemistry community that temperature rise plays a role on plasmon-enhanced photocurrents and catalytic reactions.
2) Studies where temperature rise (thermal) effects have been resolved separately from excited charge carriers (non-thermal) effects are described in literature. Nano Lett., 2018, 18 (3), pp 1714–1723 provides one clear example. Refs. 24 and 25 are two other examples. I was not able to determine what advance in insight is brought about by the current study beyond these references (or similar ones in literature). The claim in the abstract "So far, no strategies have been advanced to confidently separate the relative contribution of these two effects." therefore appears unfair. It would be more appropriate to discuss the specific findings from existing literature and then describe how the current work changes or improves upon that understanding.
3) "Novel plasmonic electrode" may also be inappropriate since electrodes consisting of plasmonic nanoparticles are now well described in literature. Some examples include the published work of Cronin, Willets, and others.

4) I had trouble following the central argument on p3-4 about the timescale of temperature rise vs. charge separation induced effects. Indeed, excited electron/hole pairs relax within the nanoparticle on the timescale of 1 ps, but this does not mean that chemistry induced by excited charge carriers has the same timescale. I believe it would be necessary to consider the timescales of charge transfer, charged intermediates, and mass transport in the local medium around the nanoparticle. Without knowing the timescale of the surface electrochemistry, it would be difficult to conclude that charge-separation-induced effects are slower than photothermally induced temperature changes in the local medium around the nanoparticle. A COMSOL simulation would allow a more rigorous analysis. The rationale for the technique described as: "Accordingly, a photoelectrochemical method with a millisecond time resolution would be able to disentangle the charge carrier from photothermal effects. Specifically, turning the light off rapidly would cause the photoelectronic effect to disappear rapidly, while the increased temperture declines more slowly" is not well-founded.
5) Fig. 4 presents tha main result: how much photocurrent is from charge separation vs. that from a photothermal contribution. But, aside from this result, which is specific to the measurements conducted here, is there a general message or model that emerges that is new and improves understanding in our community? I did not find that to be the case. Fig. c, which is stated to be a "proposed mechanism" is a textbook-level schematic used in photochemistry.

Therefore, in my view, the paper may be more suited to be reviewed and read by the analytical electrochemistry community. Some additional technical questions that the authors may want to consider:
1) In Fig. 4b, the red data points describe the photothermally induced photocurrent as a function of the excitation wavelength. Why does this plot not follow the absorption spectrum? Photothermal temperature rise would be expected to track the absorption cross-section.
2) The blue data-points describe the "photoelectronic" contribution to the photocurrent as a function of wavelength. Is there a reason why the longer-wavelength plasmon resonance mode (seen around 660 nm in Fig 1d) is not reflected in this trend.
3) The statement in the manuscript "Alternatively, the photothermal effect can increase the population

of the high vibrational excited state to promote the surface reaction" needs a better foundation. Excitation into a higher vibrational state, which is typically separated from the ground vibrational state by 0.1-0.5 eV in typical molecules would require a few 1000 K of temperature rise. Are the authors claiming such a large temperature rise under continuous wave excitation?
4) In the schematic in Fig. 4b, the potential energy surface on the left ought to be that for the ground electronic state (including donor and acceptor and solvent) prior to electron transfer and the one on the right ought to be one for the ground electronic state (including donor, acceptor, and solvent) after the electron transfer. The authors think of them/depict them as "Reagent" and "Product" electronic states, which may not be rigorously correct. The x-axis is the reaction co-ordinate.

Reviewer #2 (Remarks to the Author):

The authors use a 3-electrode setup to perform photoelectrochemistry involving a working electrode composed a plasmonic nanoparticles.
They propose this setup as a powerful means to investigate the role of photothermal processes in hot-electron induced chemical reactions.
The approach is powerful, interesting and could be the basis for numerous studies, if reproduced by the community.
I recommend this article for publication provided the authors properly address the moderate/major corrections listed below:

1- Bottom of page 3
"Based on the the accepted of SP relaxation..."
I don't understand this sentence. A word might be missing. Do the authors mean "Based on the accepted mechanism of SP relaxation"?

2- Page 4
"Whereas, heat, generated after a relaxation time of nanoseconds, leads to the increased temperature of its surroundings, and a longer time (seconds) is needed to reach thermal diffusion equilibrium (Figure 1e3)."
A nanosecond time scale is expected for a sub micrometric system, ie a single nanoparticle. Here the authors are not illuminating one nanoparticle, but an array of nanoparticles, ie a macroscopic object, hence the longer time scale of 10 s. I would clarify this sentence, which currently seems confusing for the reader with these two incompatible time scales. I would not talk about the nanosecond time scale, or explain why it is not the proper time scale for this experiment.

3- Page 4 "temperture declines" should read "temperature declines "

4- How big is the illuminated area of the electrode?

5- A schematic showing the three-electrode setup would be useful for the reader, and preferentially in the main manuscript. Please don't forget that most readers might be physicists working in optics/nanophotonics, not electrochemistry.

6a- Page 5
"the incident light can promote both oxidation reactions (e.g. reversible gold oxidation) and reduction reactions (e.g. oxygen reduction or hydrogen evolution)."
This seems to be a benefit compared to semiconductors. But what about the magnitude of the current (not only the direction). How does it compare with a semiconductor electrode?

6b- Then, as far as I understand, the effect is not as symmetric as this sentence lets think. According to Figure 2b, the current for the negative bias is 50 times larger than with a positive bias (see the factor of /50). Is that true, if yes, this non-symmetry could be mentioned while discussing this comparison with semiconductors.

7- Page 5
"which likely involve "
I would write "involves".

8- Page 6
I would explain what the Faradic current is (and put an uppercase, like 'Gaussian', 'Hermitian', ...). Again, opticians won't understand at first glance.

9- Are the authors using a stirring magnet? This should strongly influence the thermodynamics, and thus the temperature, since stirring would enhance cooling of the sample by fluid convection. Comparing/discussing experiments with and without stirring magnet would be interesting.

10- It would have been very simple to plunge a thermocouple at the vicinity of the working electrode, just after the laser is turned off (in order not to heat the thermocouple with the laser). As the presumed time scale of heating is on the order of 10 s, this leaves plenty of time to perform the measurement. This would have been a more direct manner to ascertain the occurrence of a temperature increase (see my concern #14), and validate the measurements presented in Figure 3.

11a- Eq 2, please define all the parameters. In particular, 'x' is not defined. Is it time? Why not calling it 't'?

11b- please provide a reference with eq 2 or explain where it comes from or how it was derived (if SI you prefer). Because here, it is enigmatic.

12- In principle, physical quantities such as V, I, T, ... should be italicized. This is not always the case throughout the article.

13- Figure 3d, the x-axis label reads 'P', but P is supposed to be the laser power. Do the authors rather mean 'Potential' here, like in Figure 3c?

14a- Could this slow time scale be due to a reaction-diffusion process in the liquid. Could slow diffusion of molecules/ions yield slow variations such as the ones observed in this article?

14b- Could this slow time scale also be due to the onset of a thermal-induced fluid convection, settling over a long time scale, that would affect the migration and diffusion of ions/molecules?

15- Figure 4a, it would have been meaningful to estimate the temperature increase in Figure 4a, not only the laser irradiance (see my previous comment #10).

16- Page 11
"and becomes obviously in the interband transition region."
Not sure to understand the sentence. Do you mean "...becomes obvious..."?

Reviewer #3 (Remarks to the Author):

The work by Zhan et al. describes an experimental approach to disentangle plasmon-mediated hot-

carrier and thermal effects on electrochemical reactions. The authors' approach is similar to the one described by Willets and coworkers (J Phys Chem B 2018), studying the enhancement of electrochemical conversion at a plasmonic electrode surface as a function of laser incident power and wavelength, and of temperature. The presented science is very interesting, though interpretations and conclusions not fully substantiated and the language too imprecise, partly sloppy. The analysis of the current responses and the disentanglement between hot-carrier and thermal parts is qualitative, not quantitative (as claimed in the abstract). I thus recommend to reconsider the manuscript for publication after major revisions.

The authors' main finding is the appearance of two voltammetric current response regimes: one of 'rapid response' and one of 'slow response', which the authors attribute to hot-carrier and thermal enhancement, respectively. The curves should be evaluated/fitted with respective time traces for hot-electron mediated transitions and thermal transitions to i) quantify the individual contributions (decay times tau1 and tau2, likely) and ii) substantiate the authors' assignment claim and iii) allow a comparison to classical semiconductor hot-carrier processes beyond the statement that 'the curve … has a similar line shape". Such analysis would likely allow them to indeed "quantitatively disentangle" the two effects as claimed in the abstract.

The current-response curves are different for different chemical reactions (Figs 2e for Au oxide formation, 2f for ferrocene redox cycling). How do the thermal/SRC decay times for the two - very different - surface reactions compare? Do the curves/extracted numbers make (electro)chemical sense?

While the current/time traces in Fig 2b shows a slow decay in maximum amplitude over the shown 275 seconds, indicating incomplete reversibility of the process (why?), the traces in Fig 3b look very stable over time (i.e. 50 s). How do the authors explain this difference?

What is the pzc of the system? Is the zero-current potential in Fig 2d an indicator for the pzc for the given experiment/system?

Line 210: "assuming linear non-equilibrium thermodynamics" (Why) Is this a valid assumption for near-field processes?

Line 214: What is x=0? What are the "boundary conditions"? What is A1?

Fig 3f/ line 219/220: "the excellent fit" Where does the fitting formula denoted in Fig 3f come from? From which data set are the data points in Fig 3f extracted? Error bars?

Inset in Fig 3a is too small.

In general, the error bars are missing; in Figs 2d, 3c,f and 4a error bars would be of great interest to quantitatively evaluate the observed effects. What are the error bars in Fig 4b?

Lines 238-241 From "Such photoelectrochemical methods … and photoelectrochemical processes." Leave out. Empty statements, unsubstantiated, does not belong into a results/discussion section.

Line 244ff. "The RRC showed a super-linear dependence on the incident light intensity" Exponential dependence? How to fit? Discuss, for instance, with respect to the report by Willets and coworkers who find a similar trend - but come to the opposite conclusion/ interpretation.

Fig 4b, line 248ff. The shape of the RRC and SRC curves is a very interesting observation. Why does the SRC decrease with increasing lambda indicate the "contribution of interband transitions to the photothermal effect"? What do the authors mean here? For which reaction(s) have such curves be obtained? Only for the Au oxidation?

Related to the previous comment, in line 257, the authors state that "excited carriers can transfer [their energy? or the hot electron/hole itself is transferred?] directly to the adsorbed molecule to form a charged species…" and in line 260 "the population of the high vibrational excited state to promote the surface reaction." - what are "the adsorbed molecule" and the "surface reaction" in the system studied here? What is the mechanistic picture the authors have in mind for Au oxidation? And for ferrocene reduction/oxidation? Would one not expect different preferred pathways, and thus also different enhancement mechanisms?

Line 254/255 What is a "photocurrent experiment"?

Line 262ff. "… one is better positioned to rationally design and fabricate plasmonic structures with appropriately controlled ratios of these two contributing mechanisms to gain the optimal efficiency for specific chemical reactions." It is unclear from the results, how the presented work - observing a fast and a slow current response and attributing them (qualitatively) to hot-carrier and thermal contributions to the current enhancement - would help to design improved structures. This is an unsubstantiated (empty) statement. What do the authors mean? What do they suggest concretely how to design a better structure based on the knowledge gained from their experiments?

Regarding the language, the manuscript is difficult to read already for a specialist reader as
- the use of terminology is partly imprecise (e.g. confusing potential, bias, overpotential, delta potential, delta bias, delta E; confusing laser power and laser intensity; etc),

- some uncommon terminology is used without definition (e.g. chopped light, plasmocurrent, energetic charge carriers, photoelectronic effect, plasmoelectric potential etc.)

- and the language is partly wrong so it is difficult to grasp the meaning/thought (e.g. sentences like "Whereas, heat, generated after a relaxation time of nanoseconds, leads to the increased temperature of its surroundings, and a longer time (seconds) is needed to reach thermal diffusion equilibrium." "First, upon illumination, the current was enhanced relative to the open circuit potential…" "The RRC is in good agreement with the UV/vis absorption spectrum." - there are plenty more sentences similarly imprecise like this one throughout the manuscript.).

The description of the potential jump experiments (lines 184-201) are confusing; I don't understand (beyond educated guessing) what the authors did exactly and why. I suggest to check terminology, to shorten sentences and explain the procedure step-by-step in detail.

Furthermore, it is hard to follow the scientific argumentation at many points in the text because statements just appear where insufficient explanation is given and/or no relevant literature is cited, and thoughts are not connected.

Overall recommendation: reconsider the manuscript for publication after major revisions

Dear Dr Chen,

Thank you for your email of August 28th 2018 with the referees' comments on our manuscript entitled: "Disentangling charge carrier from photothermal effects in plasmonic metal nanostructures" and for your kind suggestion on revised version. We sincerely thank three referees for their helpful and insightful comments. We have carefully answered all the referees' questions with explicit details and revised the manuscript accordingly.

Reviewers' comments:

Reviewer #1 (Remarks to the Author):

Zhan et al. describe a photoelectochemical method for separating plasmon-enhanced photocurrent into its constituent contributions from i) photothermally induced temperature rise and ii) photoinduced charge separation. The photoelectrochemical characterization is systematic - the effects of temperature and applied potential on the photocurrent are studied -- but the work is ideally suited for an analytical electrochemistry audience, which would be able to appreciate the merits of the technique used by the authors. From the standpoint of new insights or knowledge or broad appeal, the paper is rather limited.

Response 1:

We thank the referee for the positive response on "the photoelectrochemical characterization is systematic". We are regretful that the reviewer did not catch the new sights and broad appeal from the current manuscript, which indicates that we need to improve our expression so that the reviewer can understand our new findings and the broad appeal of the work.

The two key new insights from this work are listed as follows:

First, we for the first time systematically studied the photoeletrochemical behavior of a pure plasmonic metal electrode. All the reported photoelectrochemical studies are focused on the following systems: (1) metal electrode without the plasmonic effect (J. Electrochem. Soc. 1981,128, 2539-2545); (2) semiconductors and dyes (Refs. 29-34); (3) plasmonic metal-semiconductor complexes (Ref 35-40). These early fundamental studies have contributed significantly to their applicatios in photocatalytic and photovoltaic systems. Similarly, we would expect that a fundamental understanding of this photoelectrochemical behavior in pure plasmonic metals will be highly important and helpful to boost the vibrant field of plasmon-mediated chemical reactions (PMCR).

Second, we found two unique features of the photocurrent of the plasmonic electrode: (1) the plasmonic electrode can function as not only a photocathode (a source of excited electrons) but also a photoanode (delivering excited holes for an oxidation reaction), which has not been observed in semiconductors; (2) the plasmonic photocurrent can be readily separated into a rapid response current (RRC, attributed to the photoelectronic effect) and a slow response current (SRC, attributed to the photothermal effect of SPs). The photothermal and photoelectronic effects occur simultaneously in a plasmonic system and it is still challenging to separate them convincingly. Therefore, it is highly desirable to extract the key factors that influence the plasmon-mediated processes. The photoelectrochemical method appears to be an effective strategy to study these two effects separately. In addition, since the current value

directly represents the reaction rate, the photoelctrochemical method can quantitatively study the transient processes. Using this method, we for the first time quantified the wavelength and intensity response of photothermal and photoelectronic effects in a plasmonic system, separately.

Concerning the broad appeal, a simple search in ISI Web of Science using the keyword “plasmonic and surface plasmon” yielded more than 100,000 papers for the period of 1975 to 2018 (Figure R1a). Likewise, using the keyword “plasmonic/surface plasmon mediated/induced/enhanced chemical reaction/catalysis” yielded more than 2000 papers (Figure R1b). Both of the two figures present exponential growth of the two highly correlated fields. It can be clearly seen from Figure R1b that the field of PMCR is surging rapidly in recent five years due to the increasing global interests. It is highly important to separate the photothermal and photoelectronic effects for a rational design of materials for effective application of surface plasmons.

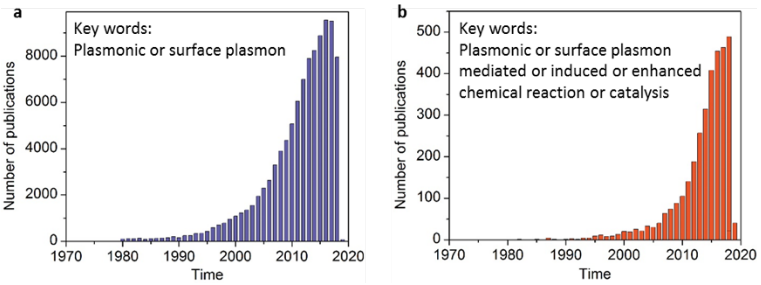

Figure R1: The number of publications related to plasmonic and PMCR searched from ISI Web of Science.

1) First, it is well appreciated in the plasmonic catalysis and plasmonic electrochemistry community that temperature rise plays a role on plasmon-enhanced photocurrents and catalytic reactions.

Response 2:

We agree with the referee that temperature rise may play a role in plasmon-enhanced catalytic reactions. Meanwhile, the excited carriers are also generated simultaneously in the plasmonic system, which also contribute to the plasmonic catalysis. Up to now, it is still a great challenge to quantitatively separate their contribution.

2) Studies where temperature rise (thermal) effects have been resolved separately from excited charge carriers (non-thermal) effects are described in literature. Nano Lett., 2018, 18 (3), pp 1714–1723 provides one clear example. Refs. 24 and 25 are two other examples. I was not able to determine what advance in insight is brought about by the current study beyond these references (or similar ones in literature). The claim in the abstract "So far, no strategies have been advanced to confidently separate the relative contribution of these two effects." therefore appears unfair. It would be more appropriate to discuss the specific findings from existing literature and then describe how the current work changes or improves upon that understanding.

Response 3:

We appreciate referee for the insightful comment and helpful suggestion. Our Response 1 has systematically demonstrated the issues of novelty and broad appeal. We will not repeat

here. We mainly list below what has been done and what can not be done with the methods proposed in the literature suggested by the reviewers:

(1) Nano Lett., 2018, 18, 1714–1723 (Ref. 27 in the revised manuscript): This paper distinguished thermal and non-thermal effects by measuring the total reaction rate and the temperature gradient of the catalyst bed to extract the effective thermal reaction rate. The authors separated the reaction bed into two parts with the known light penetration depth, i.e., the non-thermal reaction part and thermal reaction part (Figure R2). In the non-thermal reaction part, the authors assumed that various light-matter interactions (including hot-electron driven reactions, photo modification of the catalyst and enhanced near field effects) occur and the temperature of the thin layer is T1. However, even in the so-called non-thermal reaction region, the photothermal effects still exist and the thermal field is not uniform. Furthermore, the temperature distribution near the plasmonic nanostructure as a result of the photothermal effect is confined to a nanoregion of the surface and shows a gradient at the interface (Ref 9, 11), which complicates the quantitative separation of the excited carriers effect and photothermal effect. The thermal reaction part is on the millimeter scale and not on the catalysts with excited SPs.

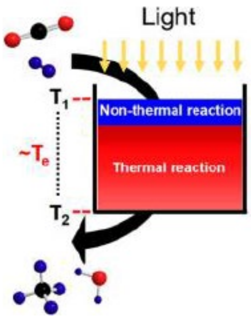

Figure R2: The schematic representation of *Nano Lett.* **18**, 1714-1723 (2018). Reprinted with permission from *Nano Lett.* **18**, 1714-1723 (2018). Copyright (2018) American Chemical Society.

(2) Ref. 25 (Ref. 26 in the revised manuscript): Willets and coworkers used SECM to elegantly isolate the roles of photoinduced reactions at the substrate and the enhanced mass transport induced by the local heating, and investigate them independently by changing the potential. However, the method was not designed to determine whether the chemical reaction is induced by the excited carriers or heating. Furthermore, SECM can only detect the product diffuses to the probe, which will be influenced by the mass transport. Therefore, it is impossible for the method to quantify the wavelength and intensity response of the photothermal and photoelectronic effects in the plasmonic system. In addition, the substrate, light spot, and SECM microelectrode probe had a different size and did not strictly match each other. The light density they used was thousands times larger than that of sunlight, which is different from the real application. Moreover, the authors mentioned in their paper that the ITO substrate used as support will influence their test: “We hypothesize that the underlying ITO substrate maybe responsible for these differences.”.

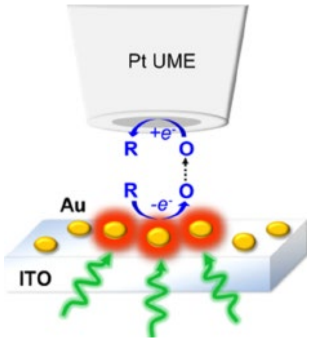

Figure R3. The schematic representation of an SECM feedback experiment described by Willets and coworkers. Reprinted with permission from *J. Phys. Chem. C* **122**, 5040-5048 (2018). Copyright (2018) American Chemical Society.

Ref. 24 (Ref. 25 in the revised manuscript). This is a work from our own group. We only reported the slow response photocurrent, and we have not yet been able to systematically investigate its origin due to the complexity of the system.

For clarity, we made the following revisions on page 1, line 22-23 in the manuscript:

"Although many efforts have been made, it is still a great challenge to confidently separate the relative contribution of these two effects."

And page 2, line 47-50

"In plasmonic systems, excited carriers and thermal effects occur simultaneously, making it hard to distinguish them confidently despite of some elegant attempts, including scanning electrochemical microscope and Raman spectroscopy.[14,25-28]"

3) "Novel plasmonic electrode" may also be inappropriate since electrodes consisting of plasmonic nanoparticles are now well described in literature. Some examples include the published work of Cronin, Willets, and others.

Response 4:

The electrode used in this study is pure metal plasmonic nanostructure with a uniform, large-area bowtie array. It differs from electrodes consisting of plasmonic nanoparticles in the following aspects.

(1) Our plasmon electrode consists of gold and quartz glass only and does not contain semiconductor or ITO. In contrast, electrodes consisting of plasmonic nanoparticles often use ITO or TiO_2 as substrate, which may influence the photoelectric response, as pointed out by Willets (Ref. 26).

(2) the as-prepared nanoparticles often have surfactants, which may lead to other unexpected effects and is difficult to achieve a good contact.

In contrast, the plasmonic bowtie array used in this work was directly fabricated on the gold film to achieve a good Ohmic contact and the electrode can even be cleaned with piranha solution to obtain a clean surface.

4) I had trouble following the central argument on p3-4 about the timescale of temperature rise vs. charge separation induced effects. Indeed, excited electron/hole pairs relax within the nanoparticle on the timescale of 1 ps, but this does not mean that chemistry induced by excited charge carriers has the same timescale. I believe it would be necessary to consider the

timescales of charge transfer, charged intermediates, and mass transport in the local medium around the nanoparticle. Without knowing the timescale of the surface electrochemistry, it would be difficult to conclude that charge-separation-induced effects are slower than photothermally induced temperature changes in the local medium around the nanoparticle. A COMSOL simulation would allow a more rigorous analysis. The rationale for the technique described as: "Accordingly, a photoelectrochemical method with a millisecond time resolution would be able to disentangle the charge carrier from photothermal effects.

Specifically, turning the light off rapidly would cause the photoelectronic effect to disappear rapidly, while the increased temperture declines more slowly" is not well-founded.

Response 5:

Referee's question is mainly on the time scale of each process in the plasmon-mediated chemical reaction system, which is still a great challenge to determine. Based on the existing literature and our understanding, we give the following estimation:

(1) On the basis of the accepted mechanism of SPs relaxation, the excited carriers generated upon illumination decay in several ps.

(2) The time scale of charge transfer is usually from hundreds of fs to ps.

(3) For charged intermediates and surface reaction, the time scale depends on the specific reactions, which was not well stated in the literature. The photocurrent induced by the excited carriers is a immediate response on the millisecond level for plasmonic metal-semiconductor complexes (Ref 29-40).

(4) For the thermal effect, heat transfers to the surroundings on a longer timescale (nanoseconds). However, a longer time, usually tens of seconds, is needed to reach the thermal diffusion equilibrium with its local surroundings. Therefore, we can conclude that the response of the photocurrent induced by the excited carriers is faster than that of the photothermal effect. Thus photoelectrochemical method with a millisecond time resolution would be able to disentangle the charge carrier from photothermal effects.

COMSOL can be used in plasmonic system for the electromagnetic field simulation or in electrochemistry for the mass transfer simulation. However, COMSOL is not able to simulate the time scale of the above processes mentioned by the reviewer. Indeed, our main purpose is to understand the unique photoelectrochemical behavior of plasmonic electrodes and try to distinguish excited carriers effect and thermal effects for plasmon-mediated chemical reaction, rather than to probe the time scale of these processes.

For clarity, we made the following revisions on page 4, line 94-103 in the manuscript. "A schematic of the photoelectrochemical process induced by the chopped light is shown in Figure 1e. On the basis of the accepted mechanism of SPs relaxation,[11,12,21] the energetic charge carriers (electrons and holes) generated upon illumination decay in several ps, which can directly impact the chemical reactions. The photocurrent induced by energetic charge carriers reaches equilibrium upon illumination in less than ms (Figure 1e2), which has been well observed and investigated in semiconductor electrodes or plasmonic metal-semiconductor complexes. Whereas, it usually takes a longer time (seconds) to reach the thermal diffusion equilibrium for the heat-induced temperature increase of the surroundings (Figure 1e3)."

5) Fig. 4 presents the main result: how much photocurrent is from charge separation vs. that from a photothermal contribution. But, aside from this result, which is specific to the measurements conducted here, is there a general message or model that emerges that is new and improves understanding in our community? I did not find that to be the case. Fig. c, which is stated to be a "proposed mechanism" is a textbook-level schematic used in photochemistry.

Response 6:

We thank the referee for the thoughtful comment. Referee’s question is mainly on the new understanding and schematic shown in Figure 4.

Based on the study of the photoeletrochemical behavior of the plasmonic metal electrode, we proposed a photoeletrochemical method to effectively study the photothermal and photoelectronic effects. This method allows one to quantify the wavelength and intensity response of these two effects separately in the plasmonic system for the first time, as shown in Figure 4. This result demonstrates the advantages of the photoelectrochemical method. It is important for designing plasmonic nanomaterials for specific applications.

It is a common practice to extend “textbook-level” model for a new systems (in this case, the PMCR system). For example, in a latest paper from Halas’s group, this model was also used (Ref 28, Science 2018, 362, 69–72). In our work, we borrowed this model to help understand the unique features of the photocurrent of the plasmonic system (the excited carriers and thermal effect both contribute to the electrochemical reaction), we improve the model by coupling these two effect together (Figure R4).

For clarity, we made the following revisions related to Figure 4c on page 11, line 273-277 in the manuscript:

“The experimental photocurrent curve demonstrates that both the photothermal and photoelectronic effects of SPs can influence the chemical reaction and response on different time scales, which can be quantitatively disentangled by the photoelectrochemical method (Figure 4c).”

And Page 11, line 284-287 in the manuscript:

“the mechanism for the photoelectronic effect and photothermal effect of SPs on the electrochemical reaction. ψ_{GR} represents the ground state of the reactant; ψ_{CT} represents the charged state of the reactant; and ψ_{GP} represents the ground state of the product.”

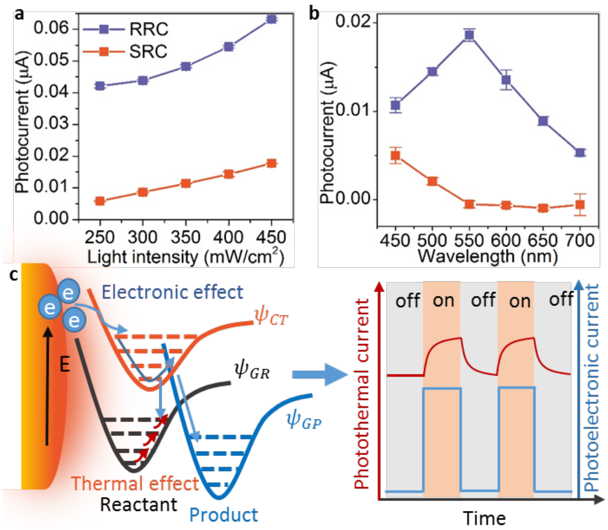

Figure R4. The revised version for Figure 4.

Therefore, in my view, the paper may be more suited to be reviewed and read by the analytical electrochemistry community. Some additional technical questions that the authors may want to consider:

1) In Fig. 4b, the red data points describe the photothermally induced photocurrent as a function of the excitation wavelength. Why does this plot not follow the absorption spectrum? Photothermal temperature rise would be expected to track the absorption cross-section.

Response 7:

Two reasons lead to the difference.

(1) The absorption spectrum shown in manuscript is in fact the extinction spectrum, which includes absorption and scattering. Photothermal effect is mainly dominated by the absorption and may not necessarily follow the lineshape of the extinction spectrum. To avoid the confusion, we change the legend of the figure from “absorption” to “extinction”.

(2) Photothermal effect and the production of energetic electrons depend on the wavelength of the incident light in plasmonic systems. For the incident light shorter than 500 nm (the interband transition from d to s band), excited electrons are created in the vicinity of the Fermi level (i.e. with low energy) which quickly thermalize. So the photothermal effect is more obvious (Ref. 11, 12, 13, 23). In contrast, when the absorbed light is resonant with the SPs (intraband region), the generated excited electrons have a higher energy. If the energetic excited carriers participate in the chemical reaction, they will not further thermalize. These are the unique features of excited carriers and photothermal effect in the PMCR system, in which these two effects interplay.

For clarity, we revised the Figure 1c.

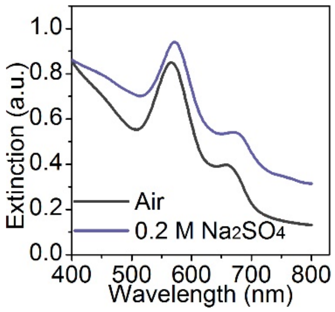

Figure R5: the UV-Vis extinction spectrum in air and in 0.2 M sodium sulfate solution

2) The blue data-points describe the "photoelectronic" contribution to the photocurrent as a function of wavelength. Is there a reason why the longer-wavelength plasmon resonance mode (seen around 660 nm in Fig 1d) is not reflected in this trend?

Response 8:

There are two reasons for the insignificant photoelectronic effect at a longer wavelength: (1) The longer-wavelength plasmon resonance mode in our work is attributed to surface plasmon polariton (SPP) of the periodic array in our previous work. So far, we have not yet seen any SPP-based plasmon chemistry. Most published works were mainly focused on localized surface plasmons. (2) The excited carriers generated under longer wavelength usually have a lower energy, which decreases the probability to induce chemical reactions.

3) The statement in the manuscript "Alternatively, the photothermal effect can increase the population of the high vibrational excited state to promote the surface reaction" needs a better foundation. Excitation into a higher vibrational state, which is typically separated from the ground vibrational state by 0.1-0.5 eV in typical molecules would require a few 1000 K of temperature rise. Are the authors claiming such a large temperature rise under continuous wave excitation?

Response 9:

Referee's question is on whether the photothermal effect of SPs can increase the population of high vibrational excited state.

By increasing the temperature, the population of vibrational excited state will increase following the Boltzmann distribution, which is reflected by the increased intensity ratio of anti-Stokes/Stokes in the conventional Raman spectroscopy. This phenomenon has been widely used to detect the temperature. It did not mean that we have to pump all the population to the excited vibrational state. Even under the excitation of SPs, the temperature rise is only about tens of degrees.

To improve the accuracy of the expression, we made the following revisions on page 11, line 271-273 in the manuscript:

"Alternatively, the photothermal effect may increase the population of activated molecules with a high kinetic energy, rotational energy, and even vibrational energy to promote the chemical reaction."

4) In the schematic in Fig. 4b, the potential energy surface on the left ought to be that for the ground electronic state (including donor and acceptor and solvent) prior to electron transfer and the one on the right ought to be one for the ground electronic state (including donor, acceptor, and solvent) after the electron transfer. The authors think of them/depict them as "Reagent" and "Product" electronic states, which may not be rigorously correct. The x-axis is the reaction co-ordinate.

Response 10:

The potential energy surface on the bottom-left is the ground electronic state of the reagent. The excited electrons transfer to the reagent to form the charged states or "transient negative ion" (Ψ_{CT}). Then the charged states can greatly enhance the reaction rates through direct relaxation to the ground state of product or use their excess energy to overcome the potential energy barriers.

For clarity, we made the following revisions related to Figure 4c on page 11, line 284 in the manuscript. Details are shown in Response 6.

Reviewer #2 (Remarks to the Author):

The authors use a 3-electrode setup to perform photoelectrochemistry involving a working electrode composed a plasmonic nanoparticles.

They propose this setup as a powerful means to investigate the role of photothermal processes in hot-electron induced chemical reactions.

The approach is powerful, interesting and could be the basis for numerous studies, if reproduced by the community.

I recommend this article for publication provided the authors properly address the moderate/major corrections listed below:

1)- Bottom of page 3

"Based on the the accepted of SP relaxation..."

I don't understand this sentence. A word might be missing. Do the authors mean "Based on the accepted mechanism of SP relaxation"?

Response 1:

Thanks for pointing out the typos. We correct the sentence into:

"On the basis of the accepted mechanism of SPs relaxation". (page 4, line 95-96)

2)- Page 4

"Whereas, heat, generated after a relaxation time of nanoseconds, leads to the increased temperature of its surroundings, and a longer time (seconds) is needed to reach thermal diffusion equilibrium (Figure 1e3)."

A nanosecond time scale is expected for a sub micrometric system, ie a single nanoparticle. Here the authors are not illuminating one nanoparticle, but an array of nanoparticles, ie a macroscopic object, hence the longer time scale of 10 s. I would clarity this sentence, which currently seems confusing for the reader with these two incompatible time scales. I would not talk about the nanosecond time scale, or explain why it is not the proper time scale for this experiment.

Response 2:

We thank the referee for the comment. For clarity, we made the following revisions on page 4, line 101-103 in the revised manuscript:

"Whereas, it usually takes a longer time (seconds) to reach the thermal diffusion equilibrium for the heat-induced temperature increase of the surroundings (Figure 1e3)."

3)- Page 4 "temperture declines" should read "temperature declines "

Response 3:

Thanks for pointing out the typos. We correct the sentence into:

"while the increased temperature declines more slowly (Figure 1e4)" (page 4, line 106).

4)- How big is the illuminated area of the electrode?

Response 4:

We added the following description on page 5, line 115 in the text:

"The area of the gold nanoelectrode array is 0.8 cm^2."

5)- A schematic showing the three-electrode setup would be useful for the reader, and preferentially in the main manuscript. Please don't forget that most readers might be physicists working in optics/nanophotonics, not electrochemistry.

Response 5:

We appreciate the suggestion. We added the following schematic to show the three-electrode setup shown as Figure 1d.

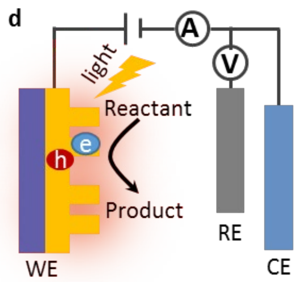

Figure R6. Schematic of the three-electrode electrochemical system used in the experiments.

6a)- Page 5

"the incident light can promote both oxidation reactions (e.g. reversible gold oxidation) and reduction reactions (e.g. oxygen reduction or hydrogen evolution)."

This seems to be a benefit compared to semiconductors. But what about the magnitude of the current (not only the direction). How does it compare with a semiconductor electrode?

Response 6:

We thank referee for pointing out the core novelty of the current work that the plasmonic electrode can function as not only a photocathode but also a photoanode. This is a unique advantage of plasmon electrodes over semiconductors or other photo-electrodes.

But we should also point out that the photocurrent of the pure metal plasmonic electrode is still small at present. As shown in Figure 2 and Figure S8, the slow response current is about 0.2 μA at 0.6 V and 13 μA at -0.6 V and the rapid response currents are about 0.2 μA at 0.6 V (reversible gold oxidation) and 0.4 μA at -0.6 V (hydrogen evolution). In comparison, the typical magnitude of photocurrent is about tens of μA or even mA under a similar condition for semiconductors. Although the photocurrent from the plasmonic electrode is still smaller than semiconductor photo-electrode, there is a plenty of room for improvement, such as designing new plasmonic nanostructures with stronger plasmonic effect or combining with other materials (including semiconductors), for such a new type of photoelectrochemical system.

For clarity, we added the following description on page 5, line 130-133.

"the current was enhanced relative to the open circuit potential (OCP, which occurs at ~ 0.2 V, Figure S4) when using both negative potential (producing a cathodic photocurrent, ~ 13 μA at - 0.6 V) and positive potential (producing an anodic photocurrent, ~ 0.4 μA at 0.6 V)."

6b)- Then, as far as I understand, the effect is not as symmetric as this sentence lets think. According to Figure 2b, the current for the negative bias is 50 times larger than with a positive bias (see the factor of /50). Is that true, if yes, this non-symmetry could be mentioned while discussing this comparison with semiconductors.

Response 7:

Yes, although the plasmonic electrode can produce both anodic and cathodic photocurrent, the photocurrents are non-symmetry at the positive and negative biases (see Figure 2d and Figure S8). Such a response is very different from semiconductor electrodes, in which the n-type semiconductor can only function as an oxidation photoelectrode and p-type only as reduction photoelectrode. Such a non-symmetry is dominated by the slow response current following the same trend as the background current. As a result, the plasmocurrent at the negative bias is much larger than that at the positive bias.

For clarity, we added the following description on page 6, line 159-161: "It should be emphasized that the plasmocurrent at the negative bias is much larger than that at the positive bias."

7)- Page 5

"which likely involve "

I would write "involves".

Response 8:

Thanks for pointing out the typos. We correct "involve" into "involves" (page 6, line 152).

8)- Page 6

I would explain what the Faradic current is (and put an uppercase, like 'Gaussian', 'Hermitian', ...). Again, opticians won't understand at first glance.

Response 9:

Sorry for the typos. "Faradic current" should be "Faradaic current". To make it clear, we made the following revision on page 6, line 163 in the manuscript. "The background current is mainly the Faradaic current due to the redox reaction on the electrode".

9)- Are the authors using a stirring magnet? This should strongly influence the thermodynamics, and thus the temperature, since stirring would enhance cooling of the sample by fluid convection. Comparing/discussing experiments with and without stirring magnet would be interesting.

Response 10:

We appreciate the referee for the suggestion. We didn't use a stirring magnet. It may be a good idea to try it in the future experiments. It is still very challenging to add a magnetic stirring system in our current experimental set-up while retainning a good signal to noise ratio during the photocurrent measurement of the plasmonic metal electrode.

10)- It would have been very simple to plunge a thermocouple at the vicinity of the working electrode, just after the laser is turned off (in order not to heat the thermocouple with the laser). As the presumed time scale of heating is on the order of 10 s, this leaves plenty of time to perform the measurement. This would have been a more direct manner to ascertain the occurrence of a temperature increase (see my concern #14), and validate the measurements presented in Figure 3.

Response 11:

The method suggested by the referee can detect the temperature of the electrolyte in the vicinity of the working electrode (millimeter range). But it is hard to measure the interfacial temperature that is essential to a photoelectrochemical system. Especially, in our photoelectrochemical experiments using a chopped incident light, there is no obvious temperature increase in the electrolyte under each cycle of "on and off" the light. More importantly, the temperature distribution in a plasmonic nanostructure is confined to a nanometer region and shows a gradient near the plasmonic structure (Ref 11). Therefore, it is still challenging to measure the local temperature of the plasmonic structure to quantitatively estimate the contribution of the photothermal effect.

11a)- Eq 2, please define all the parameters. In particular, 'x' is not defined. Is it time? Why not calling it 't'?

Response 12:

Yes, "x" is the time, we changed "*x*" to "*t*" in the revised manuscript. We made the following revision on page 9, line 222 in the manuscirpt:

"*I* is the current, A_0 and *D* are constants."

And 229-230 in the manuscirpt:

"*t* is the time"

11b)- please provide a reference with eq 2 or explain where it comes from or how it was derived (if SI you prefer). Because here, it is enigmatic.

Response 13:

We added a derivation process in SI for the eq 2. (Section 3)

12)- In principle, physical quantities such as V, I, T, ... should be italicized. This is not always the case throughout the article.

Response 14:

We italicized all of physical quantities.

13)- Figure 3d, the x-axis label reads 'P', but P is supposed to be the laser power. Do the authors rather mean 'Potential' here, like in Figure 3c?

Response 15:

Yes, the x-axis label "P" of Figure 3d is potential. We corrected this problem in Figure 3d.

14a)- Could this slow time scale be due to a reaction-diffusion process in the liquid. Could slow diffusion of molecules/ions yield slow variations such as the ones observed in this article?

Response 16:

We thank referee for the question. We have also considered the reaction-diffusion process, but we still think it is not responsible for the slow response current for the following three reasons:

(1) Reaction-diffusion commonly exists in all photoelectrochemical systems. However, no slow response current (SRC) have been observed under similar conductions for the semiconductor photoelectrode.

(2) As mentioned by the referee, the slow diffusion of molecules/ions could yield a slow response of the photocurrent. Thus the time scale of the hydrogen evolution reaction at -0.6 V involving the proton diffusion should be faster than that of the oxygen reduction at 0 V. But the experimental result is just the opposite as shown in Figure 2b.

(3) The excellent fit of the experimental SRC to the derived thermal dynamics equations also supports the conclusion that the SRC is induced by the photothermal effect.

14b)- Could this slow time scale also be due to the onset of a thermal-induced fluid convection, settling over a long time scale, that would affect the migration and diffusion of ions/molecules?

Response 17:

We thank the referee for the thoughtful question. We think the slow time scale may not be due to the onset of a thermal-induced fluid convection for the following four reasons:

(1) If the thermal-induced fluid convection plays the key role for SRC, there should be no SRC at the non-diffusion controlled potential because the reaction current at this time is not determined by the mass transfer. However, the slow process appears at both the non-diffusion controlled potential (-0.4 V\-0.6 V) and diffusion controlled potential (0.6 V).

(2) No obvious temperature increase in the electrolyte was observed in the photoelectrochemical experiments with the chopped incident light (see Response 11).

(3) The SRC follows the same trend as the background current with the applied potential. If the migration and diffusion of ions/molecules play the key role in the SRC, the trend will be affected by the types of reactants and different from the background current at different potentials.

(4) The excellent fit of the experimental SRC to the derived thermal dynamics equations also strongly supports our conclusion that the SRC is a result of the photothermal effect following plasmon decay.

But we cannot fully rule out the influence of the local thermal effect on the diffusion process, as it's also a part of photothermal effects of SPs.

15)- Figure 4a, it would have been meaningful to estimate the temperature increase in Figure 4a, not only the laser irradiance (see my previous comment #10).

Response 18:

We thank referee for the suggestion. In Figure 4a, we showed the RRC and SRC as a function of the incident light intensity, which is very important for PMCR system. As we mentioned in Response 11, the photothermal effect of a plasmonic nanostructure is confined to nanometer range and shows gradient. Thus, it is very challenging to detect the local temperature (surface or interface temperature) and the gradient of the thermal field. In fact, recently we developed a method to measure the surface temperature of plasmonic nanoparticles with strong SERS enhancement using an isocyanide molecule by measuring the frequency shift of SERS peak (J. Am. Chem. Soc. 2018, 140, 13680−13686). However, the plasmonic electrode used in this study did not show sufficient SERS enhancement to allow us to use the above method to measure the temperature.

16)- Page 11

"and becomes obviously in the interband transition region."

Not sure to understand the sentence. Do you mean "...becomes obvious..."?

Response 19:

Thanks for pointing out the typos. We correct the sentence into "and becomes obvious at the incident light shorter than 550 nm" (page 10, line 264).

Reviewer #3 (Remarks to the Author):

The work by Zhan et al. describes an experimental approach to disentangle plasmon-mediated hot-carrier and thermal effects on electrochemical reactions. The authors' approach is similar

to the one described by Willets and coworkers (J Phys Chem B 2018), studying the enhancement of electrochemical conversion at a plasmonic electrode surface as a function of laser incident power and wavelength, and of temperature. The presented science is very interesting, though interpretations and conclusions not fully substantiated and the language too imprecise, partly sloppy. The analysis of the current responses and the disentanglement between hot-carrier and thermal parts is qualitative, not quantitative (as claimed in the abstract). I thus recommend to reconsider the manuscript `for publication after major revisions.

Response 1:

We thank the referee for the comment and pointing out "the presented science is very interesting". We believe that our work has provided new insights and knowledge for plasmon chemistry (Response 1 for the first referee) and is more quantitative, which is different from Willets' in the following aspects.

(1) Willets and coworkers used SECM to elegantly isolate the roles of photoinduced reactions at the substrate and the enhanced mass transport induced by the local heating and investigate them independently by changing the potential. However, the method is not designed to determine whether the chemical reaction is induced by the excited carriers or heating. Furthermore, SECM can only detect the product diffuses to the probe, which will be influenced by the mass transport. Therefore, it is impossible for the method to quantify the wavelength and intensity response of photothermal and photoelectronic effects in the plasmonic system. In addition, the substrate, the light spot, and SECM microelectrode probe had a different size and did not strictly match each other. The density of the light they used is thousands times larger than that of sunlight. Moreover, the authors mentioned in their paper that the underlying ITO substrate used in their experiment as support will influence their test by stating: "We hypothesize that the underlying ITO substrate maybe responsible for these differences." and "We hypothesize that the variability in the ITO substrates may also affect the charge separation process."

(2) In our work, we directly detected photocurrent under a low-intensity light (on the order of solar intensity) for analysis. The photocurrent represents the reaction rate, which is beneficial to the quantitative study of transient processes. Using the photoelectrochemical method, we disentangled the photoelectronic and photothermal effects and experimentally demonstrated the excited carriers and photothermal reaction rate as a function of the incident light intensity and wavelength. We found that the wavelength dependence of the photoelectronic current tracks the SPR absorption spectrum, and shows a super-linear dependence on the incident light intensity. Whereas, the photothermal current is only linear over a certain light intensity range, and becomes obvious in the interband transition region. More importantly, the illumination area was the same as the measured area in our case, eliminating the effects of background current and uneven illumination.

For clarity, we added the following description on page 2, line 47-50 in the manuscript.

"In plasmonic systems, excited carriers and thermal effects occur simultaneously, making it hard to distinguish them confidently despite of some elegant attempts, including scanning electrochemical microscope and Raman spectroscopy."

And page 2, line 57-58.

"In addition, the value of current directly represents the reaction rate."

1) The authors' main finding is the appearance of two voltammetric current response regimes: one of 'rapid response' and one of 'slow response', which the authors attribute to hot-carrier and thermal enhancement, respectively. The curves should be evaluated/fitted with respective time traces for hot-electron mediated transitions and thermal transitions to i) quantify the individual contributions (decay times tau1 and tau2, likely) and ii) substantiate the authors' assignment claim and iii) allow a comparison to classical semiconductor hot-carrier processes beyond the statement that 'the curve … has a similar line shape". Such analysis would likely allow them to indeed "quantitatively disentangle" the two effects as claimed in the abstract.

Response 2:

We thank the referee for the thoughtful comment. We agree with the referee that it would be ideal to fit the curve with respective time traces for excited carriers mediated chemical reactions and thermal induced reactions. However, it is challenging and also inaccurate to fit the rapid response current (RRC) for the excited carrie-mediated reactions for the following reason: the time resolution of most photoelectrochemical methods is about millisecond. The photocurrent response of semiconductor electrode is on a similar time scale or even shorter, which is also the case for the photocurrent induced by excited carriers of SPs (Ref 35-40). Thus, only one or two data points can be obtained before the photocurrent reaches the steady state. Therefore, it is impossible to fit the time trace of the excited carrier-mediated reactions. This is also the case for RRC.

However, inspired by the referee, we did the following data processing. Using the fitted result shown in Figure 3f, the obtained contribution of photothermal effect in the first 0.05 s (attributed to the photoelectronic effect) is less than 0.5 % and is negligible. The influence of the photothermal effect on the current may take a relatively long time to reach equilibrium (tens of seconds). So the photoelectrochemical method with a millisecond time resolution would be able to disentangle the excited carrier from photothermal effects. Since the current value directly represents the reaction rate, the photoelctrochemical method can quantitatively study these two processes.

For clarity, we made the following revision on page 6, line 153-157 in the manuscript:

"Similar photocurrent response to RRC has been observed and investigated in semiconductor electrodes and plasmonic metal-semiconductor complexe,[29-34] and has been attributed to excited carriers. Therefore, we assign the RRC observed in the plasmonic electrode to excited carriers.[35-40]"

and 161-166 in the manuscript:

"Different from the opposite polarity of RRC to the background current at 0 V, the SRC follows the same polarity and trend as the background current (the dark current). The background current is mainly the Faradaic current due to the redox reaction on the electrode, indicating the SRC is related to the electrochemical reaction (Figure S8, S9). For example, at 0.2 V (near the OCP), although the RRC is very obvious, the SRC is almost zero (Figure 2c)."

2) The current-response curves are different for different chemical reactions (Fig 2e for Au oxide formation, 2f for ferrocene redox cycling). How do the thermal/SRC decay times for the two - very different - surface reactions compare? Do the curves/extracted numbers make (electro)chemical sense?

Response 3:

We thank the referee for the carefully observation. It will be very meaningful but up to now still very difficult to associate SRC decay times with specific reactions. We found the SRC follows the same trend as the background current including the current polarity and magnitude and the SRC curves at -0.4 V and -0.6 V are different for different background currents (reaction rates) even for the same chemical reaction.

3) While the current/time traces in Fig 2b shows a slow decay in maximum amplitude over the shown 275 seconds, indicating incomplete reversibility of the process (why?), the traces in Fig 3b look very stable over time (i.e. 50 s). How do the authors explain this difference?

Response 4:

We thank the referee for the question. The main difference in Fig 2b and Fig 3b is the SRC. For the typical potential step curves in Fig 3b, there are no photothermal effect. The slow decay is mainly from the SRC. If there is the photothermal effect, it is difficult to allow the system to completely return to the initial state during each chopped cycle. As shown in Figure R7, when we increased the frequency of the chopped-light, the decay will be obvious.

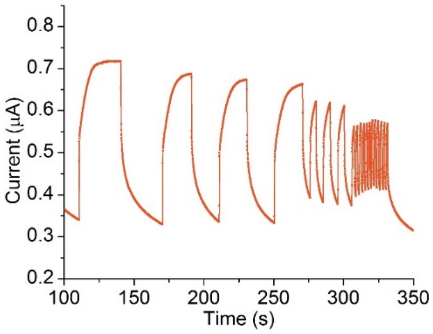

Figure R7: The photocurrent of Au nanoelectrode array at 0.6 V under the illumination of the chopped light.

4) What is the pzc of the system? Is the zero-current potential in Fig 2d an indicator for the pzc for the given experiment/system?

Response 5:

We thank the referee for the question. The pzc (the potential when the electrical charge of the surface is zero) of the gold electrode in Na_2SO_4 electrolyte is about -0.05 V (vs SCE). (J. Phys. Chem. 1967, 71, 792-797; Modern electrochemistry 2A, fundamentals of electrodics, 2nd ed., Kluwer Academic/Plenum Publishers, New York, p 862). The zero-current potential of SRC in Fig 2d is the open circuit potential (about 0.2 V) instead of pzc. However, the zero-current potential of RRC is close to pzc of the gold electrode (Figure R8). The reason for this is still unclear, which requires further investigation.

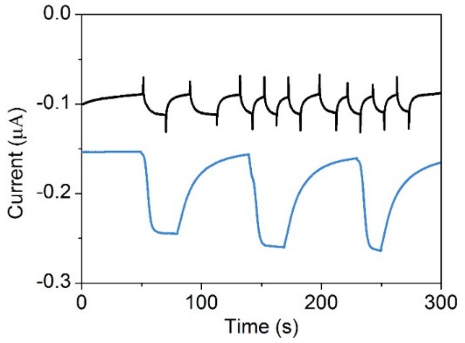

Figure R8. The current-time curves of the Au nanoelectrode array at 0 V and -0.05 V.

5) Line 210: “assuming linear non-equilibrium thermodynamics” (Why) Is this a valid assumption for near-field processes?

Response 6:

We thank the referee for the question. Yes, this theory is commonly used for the heat diffusion in plasmonic systems, such as in Ref. 11 or other works like Nano Lett. 2014, 14, 4640-4645 and J. Phys. Chem. C 2007, 111, 3636-3641.

For clarity, we made the following revision on page 9, line 224 and 226 in the manuscript: “The system is an open system being heated at a constant light intensity. The thermal dynamics on the basis of the linear non-equilibrium thermodynamics yields the following expression:”

6) Line 214: What is x=0? What are the “boundary conditions”? What is A1?

Response 7:

“*x*” presents the time. At the beginning, $x = 0$ and $T = T_0$. For clarity, we changed “*x*” to “*t*” in the revised manuscript. The detailed derivation process is added in SI (Section 3). Eq. 2 in the original version of manuscript is now shown as follows:

$$T = A_1 e^{B - \frac{ak}{cl}t} + \frac{Pl}{ak} + T_0$$

A_1 and B are constants. Here we use "l" to replace “*b*”.

Eq. 2 can also be expressed as

$$T = Ae^{-\frac{ak}{cl}t} + \frac{Pl}{ak} + T_0$$

where $A = A_1 e^B$

A is constants that is determined from boundary conditions. Assuming $T = T_0$ at $t = 0$, we obtain equation:

$$A = -\frac{Pl}{ak}$$

in which, *A* is negative because the energy input by the incident light is positive.

7) Fig 3f/ line 219/220: “the excellent fit” Where does the fitting formula denoted in Fig 3f come from? From which data set are the data points in Fig 3f extracted? Error bars?

Response 8:

We thank the referee for the question. The details were shown on page 9 from line 221-236 in the manuscript. Based on Equation 1 and 2 in our manuscript, we can get the following expression:

$$I = A_0\left(Ae^{-\frac{ak}{cl}t} + \frac{Pl}{ak} + T_0\right) + D$$

which can be further expressed as:

$$I = I_0 + Be^{-\frac{t}{b}}$$

This is the fitting formula.

The data in Figure 3f is from Figure 2b at 0.6 V from 200 s to 240 s. The SRC of every cycle can be fitted with the above equation, regardless of the applied bias or chemical reaction.

We obtained the curve via a long time continuous tracking of the current, which does not allow us to draw error bars experimentally. In fact, the change of current with time can better reflect the fluctuation and with the chopped light can reflect the reversibility. We just followed the common practice widely adopted in the field. Most of the current-time curves and temperature-time curves do not give the error bar. Figure R9 shows some typical current-time curves and temperature-time curves copied from literatures.

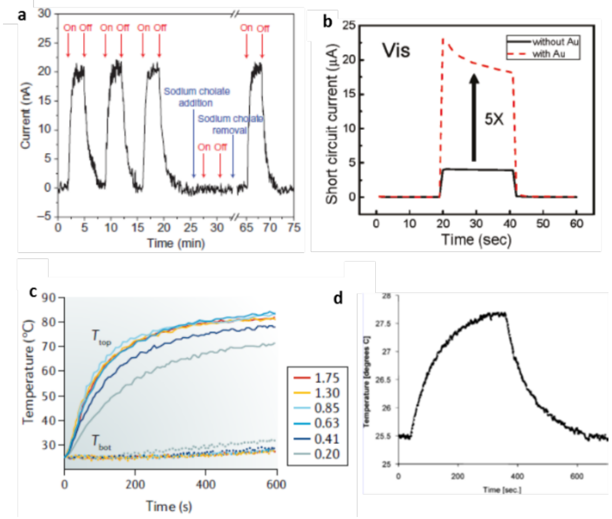

Figure R9. The typical current-time curves and temperature-time curves copied from the literatures: (a) Nature Chemistry, 2010, 2, 929–936; (b) Nano Lett. 2011, 11, 1111–1116; (c) Nano Lett. 2014, 14, 4640-4645; (d) J. Phys. Chem. C 2007, 111, 3636-3641. (in Willets' work, Ref 29-40, Nature Catalysis, 2018, 1, 412–420; Nature Energy, 2017, 2, 17045, they also did not give the error bar). Part (a) is reprinted with permission from *Nat. Chem.*, **2**, 929–936 (2010). Copyright (2010) Springer Nature Limited. Part (b) is reprinted with permission from *Nano Lett.* **11**, 1111–1116 (2011). Copyright (2011) American Chemical Society. Part (c) is reprinted with permission from *Nano Lett.* **14**, 4640–4645 (2014). Copyright (2014) American Chemical Society. Part (d) is reprinted with permission from *J. Phys. Chem. C.* **111**, 3636–3641 (2007). Copyright (2007) American Chemical Society.

8) Inset in Fig 3a is too small.

Response 9:

We thank the referee for the suggestion. For clarity, we have redrawn Figure 3, which is presented below and highlighted on page 9.

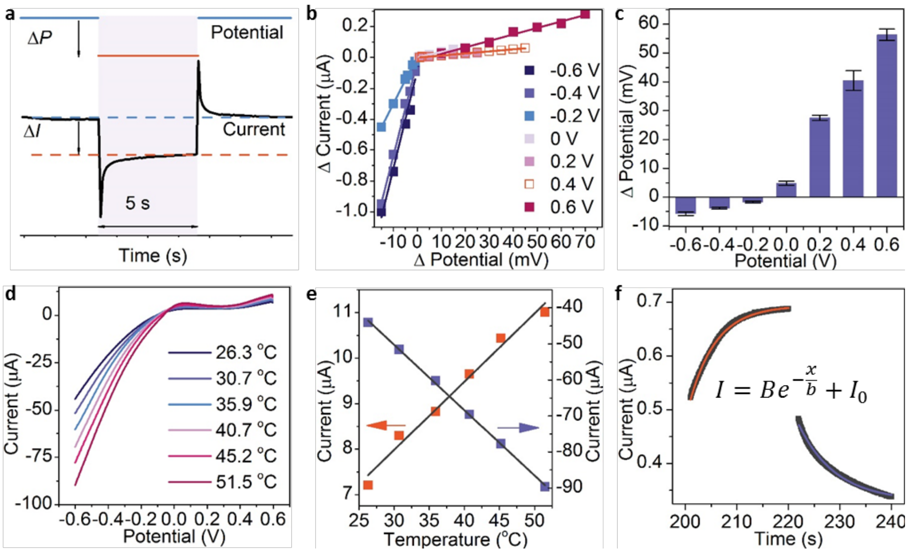

Figure R10. The revised Figure 3.

9) In general, the error bars are missing; in Figs 2d, 3c,f and 4a error bars would be of great interest to quantitatively evaluate the observed effects. What are the error bars in Fig 4b?

Response 10:

Thanks for the suggestion.We added the error bars of Figure 2d and 3c, which are given below and highlighted on page 7 and 9 in the manuscrpt. For Figure 3f, we obtained the curve through a long time continuous tracking test, which does not allow us to draw error bars experimentally for Figs. 3f, as shown in Response 8. For Figure 4, we obtained the photocurrent-time curves at different light intensities or wavelengths and extracted the values of RRC and SPR from the curves. We do show error bars to reflect the fluctuation of current in Figure 4a and 4b.

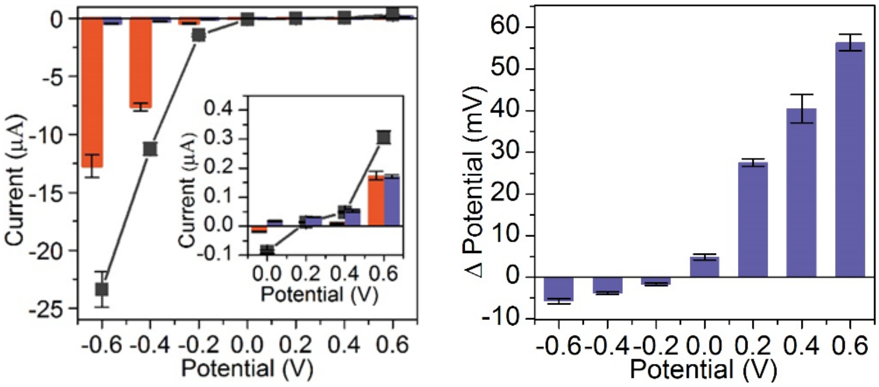

Figure R11. The revised Figure 2d and Figure 3c.

10) Lines 238-241 From “Such photoelectrochemical methods … and photoelectrochemical processes.” Leave out. Empty statements, unsubstantiated, does not belong into a results/discussion section.

Response 11:

We prefer to keep this sentence as a connection between two sections. In the previous section of the manuscript, we proposed that the photoelectrochemical method can be a strategy to study excited carriers and photothermal effects separately. In this section, we used this method to experimentally demonstrate the contributions of the two effects for chemical reaction separately. With the understanding of which effect is important for a specific reaction, one can then rationally design reaction systems that can most effectively utilize SP excitation.

For clarity, we made the following revision on page 10, line 251-253 in the manuscript. "Such photoelectrochemical methods allow us to distinguish between the plasmon-mediated photoelectronic and photothermal effects to quantitatively investigate contributions of these two effects for chemical reactions."

11) Line 244ff. "The RRC showed a super-linear dependence on the incident light intensity" Exponential dependence? How to fit? Discuss, for instance, with respect to the report by Willets and coworkers who find a similar trend - but come to the opposite conclusion/ interpretation.

Response 12:

We thank the referee for the questions. Although the experimental data can be fitted by exponential function, it can be better fitted by the polynomial function considered to be an important reaction behavior of excited carriers induced by SPs (Ref. 5, 18, 52). A comparison of the fitting result is given in Figure R7

Our work is different from Willets', which has been detailed in the Response 1. In brief, Willets and coworkers focused on isolating the roles of photoinduced reactions at the substrate and enhanced mass transport due to local heating independently. Thus, they attributed the non-linear dependence of reaction rate on the incident light intensity to photothermal effect.

On the other hand, we provided a method to investigate the excited carriers effect and local heating for chemical reactions separately. Thus we can quantify the dependence of reaction rates induced by photothermal or photoelectronic effect on the light intensity and wavelength in plasmonic systems. Based on the photoelectrochemical experiments, we found that RRC showed a super-linear dependence on the incident light intensity and the current shows a linear dependence on the temperature in a certain temperature range.

For clarity, we added Figure R12 in the Supporting information as Figure S14.

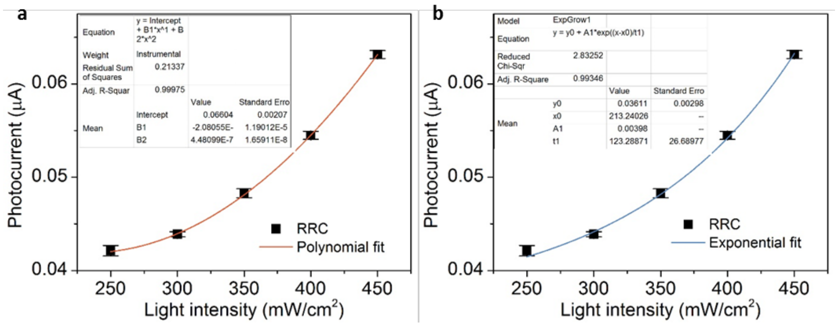

Figure R12. The polynomial (a) and exponential (b) fitting of RRC as a function of the incident light intensity

And page 10, line 256-258.

"The RRC shows a super-linear dependence on the incident light intensity, which was fitted by polynomial (Figure S14), in contrast to the linear dependence of the SRC."

12) Fig 4b, line 248ff. The shape of the RRC and SRC curves is a very interesting observation. Why does the SRC decrease with increasing lambda indicate the "contribution of interband transitions to the photothermal effect"? What do the authors mean here? For which reaction(s) have such curves be obtained? Only for the Au oxidation?

Response 13:

In our results, we found the SRC became obvious under illumination with the wavelength shorter than 550 nm (allow the excitation of the *d* to *s* interband transition). For the interband transition, excited electrons are created in the vicinity of the Fermi level (i.e. with low energy) and can quickly thermalize. So the photothermal effect is more obvious for the interband transition. In contrast, when the absorbed light is resonant with the SPs in the intraband transition region, excited electrons can be efficiently generated with a high energy (Ref. 11, 12, 13, 23). If the energetic excited carriers participate in the chemical reaction, they will not further thermalize.

In this work, we just used the Au oxidation as a model to investigate their dependences on the intensity and wavelength of the incident light. We believe that other reactions may also follow a similar curve. It should be emphasized that different reactions may require different ratios of photothermal and photoelectronic effects, indicating the importance of distinguishing the contribution of photoelectronic from photothermal effects.

For clarity, we made the following revision on page 10, line 263-267 in the manuscript. "In contrast, the SRC increases with the decreasing wavelength and becomes obvious at the incident light wavelength shorter than 550 nm (corresponding to the interband transition from d to s band), which indicates the interband transition makes the major contribution to the photothermal current."

13) Related to the previous comment, in line 257, the authors state that "excited carriers can transfer [their energy? or the hot electron/hole itself is transferred?] directly to the adsorbed molecule to form a charged species…" and in line 260 "the population of the high vibrational excited state to promote the surface reaction." - what are "the adsorbed molecule" and the "surface reaction" in the system studied here? What is the mechanistic picture the authors have in mind for Au oxidation? And for ferrocene reduction/oxidation? Would one not expect different preferred pathways, and thus also different enhancement mechanisms?

Response 14:

What we meant in the sentence "The excited carriers can transfer directly to the adsorbed molecule" is the transfer of charge (excited electrons or holes) from the plasmonic electrode to the adsorbed molecules to promote the reaction, which is commonly accepted in the plasmon-mediated chemical reactions (PMCR) (Ref. 1, 3, 5, 9, 10, 12, 13, 20, 45). If the processes are determined by the energy transfer mentioned by the reviewer, it would require the overlap of the plasmonic spectrum and the absorption spectrum of the reactants (Ref. 4).

The remained four questions focus on the reaction mechanisms. Understanding of the reaction mechanism is important, yet very challenging, which may require the molecular level techniques, such as in situ Raman or infrared. However, both techniques have their limit (frequency range or sensitivity) in studying the present system. On the other hand, although the photoelectrochemistry technique introduced in this work is powerful in disentangling the two effects, it lacks the molecular level information. Up to now, it is still difficult to obtain molecular

level evidences for the PMCR process. In our previous studies, we have tried to use SERS to monitor the reaction process of PMCR (Ref. 19, 20, 45), such as the selective oxidation of p-aminothiophenol (PATP) to p,p′-dimercaptoazobenzene (DMAB). This system has become one of the most widely studied PMCR systems. However, there are still some processes, i.e. how the oxygen was activated, lacking experimental evidence.

For clarity, we made the following revision on page 10, line 254-256 in the manuscript:

"The dependences of the plasmonic photocurrents on the light intensity and wavelength at 0.6 V free of oxygen are shown in Figures 4a and 4b (More details are shown in Figure S12)."

and on page 11 line 269-273 in the manuscript.

"The excited carriers can directly transfer to reactants to form a charged species to greatly enhance the reaction rates, or use their excess energy to overcome the energy barriers required for the reaction.[1,5,9,10,13] Alternatively, the photothermal effect can increase the population of activated molecules with a high kinetic energy, rotational energy and even vibration energy to promote the chemical reaction."

14) Line 254/255 What is a "photocurrent experiment"?

Response 15:

For clarity, we made the following revision on page 11, line 273-275 in the manuscript:

"The experimental photocurrent curve demonstrates that both the photothermal and photoelectronic effects of SPs can influence the chemical reaction"

15) Line 262ff. "… one is better positioned to rationally design and fabricate plasmonic structures with appropriately controlled ratios of these two contributing mechanisms to gain the optimal efficiency for specific chemical reactions." It is unclear from the results, how the presented work - observing a fast and a slow current response and attributing them (qualitatively) to hot-carrier and thermal contributions to the current enhancement - would help to design improved structures. This is an unsubstantiated (empty) statement. What do the authors mean? What do they suggest concretely how to design a better structure based on the knowledge gained from their experiments?

Response 16:

We thank the referee for the question. In the plasmonic system, the photoelectronic effect and photothermal effect occur simultaneously and are challenging to separate them for an effective exploration of the mechanism and development of catalyst for PMCR. In this work, we used photoelectrochemical method to quantitatively disentangle the photoelectronic and photothermal effects to extract the key factors influencing the specific chemical reaction, which may allow us rationally design the structure. For example, if the excited carriers make the main contribution, we should design the plasmonic nanostructure that can transfer the solar energy to excited carriers more efficiently, vice versa.

For clarity, we made the following revision on page 11, line 277-281 in the manuscript. "Distinguishing the contribution of the photoelectronic effect from the photothermal effect and extracting the key factors that influence the PMCR, we can be better positioned to rationally design plasmonic structures with appropriately controlled ratios of these two contributing effects to gain the optimal efficiency for specific chemical reactions."

16) Regarding the language, the manuscript is difficult to read already for a specialist reader as the use of terminology is partly imprecise (e.g. confusing potential, bias, overpotential, delta potential, delta bias, delta E; confusing laser power and laser intensity; etc),

Response 17:

To avoid such a confusion, we made some revisions. We changed all the “bias” and “overpotential” to “potential” for a consistent expression. “delta potential”, “delta bias” and “delta E” represent the potential change, we unified them as “delta potential”. In the paper, “light power” and “light intensity” have the same meaning, the unit is mW/cm^2. we unified them as “light intensity”.

17) - some uncommon terminology is used without definition (e.g. chopped light, plasmocurrent, energetic charge carriers, photoelectronic effect, plasmoelectric potential etc.)

Response 18:

We thank the referee for the comment. In fact, all the terms other than plasmocurrent have been used in the literature, including chopped light (Journal of Applied Physics, 1976, 47, 64; Energy Environ. Sci., 2011, 4, 958-964), energetic charge carriers (Ref. 10), photoelectric effec t (Ref.12, 13), photoelectronic effect (Appl. Phys. Lett. 1977, 31, 292; Appl. Phys. Lett. 1978, 33, 353), plasmonelectric potential (Ref. 48). We have also defined plasmocurrent as the photocurrent measured following the photoexcitation of the plasmonic electrode. For clarity, we changed “plasmocurrent” to “plasmonic photocurrent” to make it readily understood by readers.

18)- and the language is partly wrong so it is difficult to grasp the meaning/thought (e.g. sentences like “Whereas, heat, generated after a relaxation time of nanoseconds, leads to the increased temperature of its surroundings, and a longer time (seconds) is needed to reach thermal diffusion equilibrium.” “First, upon illumination, the current was enhanced relative to the open circuit potential…” “The RRC is in good agreement with the UV/vis absorption spectrum.” - there are plenty more sentences similarly imprecise like this one throughout the manuscript.).

Response 19:

One of our co-authors, Martin Moskovits is a native English speaker and has made great efforts to make the manuscript easily understood by a broad readership.

19) The description of the potential jump experiments (lines 184-201) are confusing; I don’t understand (beyond educated guessing) what the authors did exactly and why. I suggest to check terminology, to shorten sentences and explain the procedure step-by-step in detail. Furthermore, it is hard to follow the scientific argumentation at many points in the text because statements just appear where insufficient explanation is given and/or no relevant literature is cited, and thoughts are not connected.

Response 20:

We thank the referee for the comments. We made corresponding revisions for Figure 3 and the related description on page 8, line 196-201:

“It has been discovered that SPs can generate the electric potential under the light illumination, namely the plasmoelectric potential, which converts the optical energy into the electrical energy.[51] Our experiments show that the illumination of the Au nanoelectrode array can enhance the electrochemical reaction and lead to a RRC similar to the potential-step (*i*-*t*)

curve. The similarity suggests that we can use the potential step method to mimic the RRC process."

Reviewers' comments:

Reviewer #1 (Remarks to the Author):

I appreciate the authors’ attempts and efforts to respond to my queries and concerns. I read the response letter carefully and evaluated the revised manuscript. I am afraid that I have become even more convinced that the paper presents work that is incremental and narrow in scope and technically flawed. I apologize for having an unfavorable opinion, but I feel I would be doing disservice to the community and to the authors if I do not voice my concerns again. I care about the fundamentals of plasmonic electrochemistry, but my understanding did not grow after reading the paper. I provide a critique of the author’s responses and revisions. In my opinion, the paper itself has not undergone any improvement and central flaws remain.

1) I raised a question about the claims of novelty in the paper. In response, the authors appear to have moved the goal post. Now the authors state that the novel aspect is not a plasmonic metal electrode but a pure plasmonic metal electrode. They acknowledge that plasmonic metal electrodes have been designed and studied, but the difference is that these electrodes were supported on a semiconductor. Here is a direct quote of the author’s response: “First, we for the first time systematically studied the photoeletrochemical behavior of a pure plasmonic metal electrode. All the reported photoelectrochemical studies are focused on the following systems: (1) metal electrode without the plasmonic effect (J. Electrochem. Soc. 1981,128, 2539-2545); (2) semiconductors and dyes (Refs. 29-34); (3) plasmonic metal-semiconductor complexes (Ref 35-40).”
I fail to see how this incremental difference is going to be relevant to a broad audience. Yes, to a narrow group of researchers, that difference may matter, so why not target the work to specific experts?

2) The authors also claim that their study has merits because the number of papers related to plasmonic chemistry is growing. First-of-all, the increase in the number of papers does not provide any evidence for the value or merits of the author’s work. Secondly, the fact that there are so many papers already published would suggest that the findings of a very specific nature are best-suited to a specialized journal.

3) The authors say that the central motivation of the work is novel: “Using this method, we for the first time quantified the wavelength and intensity response of photothermal and photoelectronic effects in a plasmonic system, separately.” This claim does disservice to my colleagues in the field who have quantified these effects, in some cases as a function of wavelength and intensity. I list selected papers from ones I know:
https://pubs.acs.org/doi/10.1021/acsnano.8b03929
https://pubs.acs.org/doi/abs/10.1021/acs.nanolett.7b04776
https://pubs.rsc.org/en/content/articlelanding/2018/fd/c8fd00057c#!divAbstract
https://pubs.acs.org/doi/abs/10.1021/acs.nanolett.6b01373
https://pubs.acs.org/doi/10.1021/acs.jpcc.7b12080
The very theme of these papers was to distinguish photothermal and photocarrier effects.

4) The authors themselves published a paper in 2015 called “Quantitative detection of photothermal and photoelectrocatalytic effects induced by SPR from Au@Pt nanoparticles”. The current work is an increment over this published paper.

5) The assignment of the slow and fast responses to photothermal and photocarrier effects do not have any technical or evidentiary basis. I raised this question and the authors have not been able to answer it. When I read their response, I recognized that there is some confusion about this point, which continues to persist. The authors say “For the thermal effect, heat transfers to the surroundings on a longer timescale (nanoseconds). However, a longer time, usually tens of seconds, is needed to reach the thermal diffusion equilibrium with its local surroundings…” Likewise, the response to

photocarrier processes can take a long time to reach steady-state. While e-ph relaxation times are on the order of 1 ps, the timescale to reach a steady-state in response to the photocarriers will depend on the timescale of electrochemical reactions and diffusion of charged intermediates. This timescale can be in the microseconds, milliseconds, or even seconds range. It simply depends on the timescale of reactions and diffusion just like the timescale for thermal steady-state to be reached in the medium depends on thermal conductivities and heat transfer coefficients. Therefore, the interpretation "...photoelectrochemical method with a millisecond time resolution would be able to disentangle the charge carrier from photothermal effects." is not evidence-based but guess-work-based and a bit naive. This argument is the central piece in their analysis, so the entire paper rests on shaky ground, in my opinion.

6) I had asked "In Fig. 4b, the red data points describe the photothermally induced photocurrent as a function of the excitation wavelength. Why does this plot not follow the absorption spectrum? Photothermal temperature rise would be expected to track the absorption cross-section." The response was "The absorption spectrum shown in manuscript is in fact the extinction spectrum, which includes absorption and scattering. Photothermal effect is mainly dominated by the absorption and may not necessarily follow the line-shape of the extinction spectrum." I have experience measuring such spectra. The line-shape and peak maximum location (550 nm) of the extinction spectrum is similar to what is expected for the absorption spectrum in this case. It would have been quite simple for the authors to measure the pure absorption spectrum by diffuse reflectance and they would find that it is quite similar in shape to the extinction spectrum. But the authors did not measure the absorption spectrum. So, really the authors' response is, in my opinion, equivalent to sweeping the issue under the rug. So, once again, let me state the problem: the photothermal temperature rise does not track the spectrum (extinction or absorption), which indicates that the authors are not measuring the photothermal temperature rise. I hope the authors take this issue seriously.

7) One of the author's last response to me states "The excited carriers generated under longer wavelength usually have a lower energy, which decreases the probability to induce chemical reactions." This is demonstrably incorrect. Several groups have shown chemical reactions induced by excitation at longer wavelengths (650 nm, for instance which matches with the longer-wavelength resonance in the authors' work): e.g.

https://pubs.acs.org/doi/full/10.1021/acscentsci.7b00122

I feel that the authors are choosing to ignore published evidence to justify ignoring an important anomaly in their results.

8) MINOR POINT: Fig. 4b is still incorrect. In a rigorous electron transfer theory depiction, the initial potential energy surface is that of all reagents (including solvent) prior to electron transfer. The final potential energy surface is that of all reagents (including solvent) post-electron transfer. The authors label the initial potential energy surface as "reactant" and the final potential energy surface as "product", which is a misunderstood version of potential energy surfaces mean in electron transfer. Given the other weakness of the paper, this point is relatively minor, especially because this mistake is common, but as a physical chemist I felt it obligatory to point out the issue. The x-axis is the reaction (configurational) co-ordinate, which the authors choose not to label.

Reviewer #2 (Remarks to the Author):

The authors properly answered my concerns.

Reviewer #3 (Remarks to the Author):

The authors have addressed all comments in a satisfactory way and the manuscript has improved substantially. I recommend publication as it is now.

Reviewers' comments:

Reviewer #1 (Remarks to the Author):

I appreciate the authors' attempts and efforts to respond to my queries and concerns. I read the response letter carefully and evaluated the revised manuscript. I am afraid that I have become even more convinced that the paper presents work that is incremental and narrow in scope and technically flawed. I apologize for having an unfavorable opinion, but I feel I would be doing disservice to the community and to the authors if I do not voice my concerns again. I care about the fundamentals of plasmonic electrochemistry, but my understanding did not grow after reading the paper. I provide a critique of the author's responses and revisions. In my opinion, the paper itself has not undergone any improvement and central flaws remain.

1) I raised a question about the claims of novelty in the paper. In response, the authors appear to have moved the goal post. Now the authors state that the novel aspect is not a plasmonic metal electrode but a pure plasmonic metal electrode. They acknowledge that plasmonic metal electrodes have been designed and studied, but the difference is that these electrodes were supported on a semiconductor. Here is a direct quote of the author's response: "First, we for the first time systematically studied the photoeletrochemical behavior of a pure plasmonic metal electrode. All the reported photoelectrochemical studies are focused on the following systems: (1) metal electrode without the plasmonic effect (J. Electrochem. Soc. 1981,128, 2539-2545); (2) semiconductors and dyes (Refs. 29-34); (3) plasmonic metal-semiconductor complexes (Ref 35-40)."
I fail to see how this incremental difference is going to be relevant to a broad audience. Yes, to a narrow group of researchers, that difference may matter, so why not target the work to specific experts?

2) The authors also claim that their study has merits because the number of papers related to plasmonic chemistry is growing. First-of-all, the increase in the number of papers does not provide any evidence for the value or merits of the author's work. Secondly, the fact that there are so many papers already published would suggest that the findings of a very specific nature are best-suited to a specialized journal.

Response 1:
The general comments and the first two comments of the reviewers are indeed related to the novelty and the new insights of this work. We respond to them here together:
We do agree with the Reviewer 1 that "*the increase in the number of papers does not provide any evidence for the value or merits of the author's work.*" While we gave the two figures to reflect the current trends of the field, we provided a more important statement following the figures *"It can be clearly seen from Figure R1b that the field of PMCR is surging rapidly in recent five years due to the increasing global interests. It is highly important to separate the photothermal and photoelectronic effects for a rational design of materials for effective application of surface plasmons."* In 2010, we observed the plasmon-induced oxidation of p-aminothiophenol and thereafter we are among the very few groups who are able to explore the

reaction mechanisms. Therefore, we as well as a lot of other groups understand the photothermal and photoelectronic effects occur simultaneously in a plasmonic system and understand how important it is to convincingly separate these two effects for a better design of plasmonic photocatalyst for plasmonic chemistry systems. We clearly described these challenges in our recent review paper (Nature Reviews Chemistry, 2018, 2, 216-230).

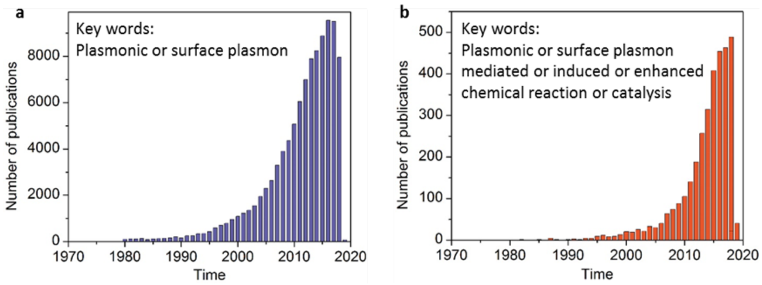

Figure R1. Publications related to plasmonics or surface plasmon (a) and related to plasmonic or surface plasmon mediated or induced or enhanced chemical reaction or catalysis (b).

In the past 50 years, the photoelectrochemical behaviors of semiconductors and dyes have been intensively studied, which provides a fundamental understanding to guide their photocatalytic and photovoltaic applications (Refs. 29-34). The photoelectrochemical processes of the plasmonic systems are less studied, and most of them were focused on the photocurrent response in plasmonic metal-semiconductor complexes (Refs. 35-40). However, the presence of semiconductors complicates the system and prevents a comprehensive understanding of photoelectrochemical behavior of the excitation and relaxation of surface plasmons. Therefore, it is highly important to study the photoelectrochemical behavior of pure plasmonic metals first, which can help the rational design of the highly efficient photocatalysis and reveal the plasmon-mediated chemical reactions (PMCR).

In this work, we for the first time systematically studied the photoeletrochemical behavior of a pure plasmonic metal electrode and **found two unique features that have never been reported in the photocurrent response of plasmonic electrodes**: (1) the plasmonic electrode can function as not only a photocathode (a source of excited electrons) but also a photoanode (delivering excited holes for an oxidation reaction), which has never been observed in semiconductors and plasmon-semiconductor complexes; and (2) the plasmonic photocurrent can be readily separated into a rapid response current (RRC) and a slow response current (SRC), which have been convincingly attributed by us to be associated with the photoelectronic effect and the photothermal effect of SPs, respectively. Indeed, these two effects play distinctively different role in PMCR. We offer in this manuscript a photoelectrochemical method that is surprisingly simple and adoptable to allow convenient separation of the photoelectronic and photothermal effects. Since the current directly represents the reaction rate and this method can quantitatively study the transient processes and we for the first time quantified the wavelength and intensity response of photothermal and photoelectronic effects in a plasmonic system.

Therefore, (1) the use of RRC and SRC in photoelectrochemical response to quantitatively separate the photoelectronic and photothermal effect and (2) the plasmonic electrode can serve both as anode and cathode endow the novelty and significance of this work to be published in this journal with a wide visibility. In this way,

the researcher in the field can use this method to understand and guide their design of plasmonic systems and promote the healthy development of the field. Additionally, our work demonstrated that a pure metal with plasmonic property can be used as a new type of photoelectrochemical material.

For clarity, we made the following revision on page 3, line 61 in the manuscript:
"But the presence of semiconductors complicates the system and prevents a comprehensive understanding of photoelectrochemical behavior of SPs. Therefore, it is highly important to study this behavior of pure plasmonic metals first, which not only provides a fundamental understanding of SPs but also will be of significant importance for the various applications of SPs."

3) The authors say that the central motivation of the work is novel: "Using this method, we for the first time quantified the wavelength and intensity response of photothermal and photoelectronic effects in a plasmonic system, separately." This claim does disservice to my colleagues in the field who have quantified these effects, in some cases as a function of wavelength and intensity. I list selected papers from ones I know:
https://pubs.acs.org/doi/10.1021/acsnano.8b03929
https://pubs.acs.org/doi/abs/10.1021/acs.nanolett.7b04776
https://pubs.rsc.org/en/content/articlelanding/2018/fd/c8fd00057c#!divAbstract
https://pubs.acs.org/doi/abs/10.1021/acs.nanolett.6b01373
https://pubs.acs.org/doi/10.1021/acs.jpcc.7b12080
The very theme of these papers was to distinguish photothermal and photocarrier effects.

Response 2:
We thank the referee for the comment. The Reviewer 1 listed five papers following the comment: *"Using this method, we for the first time quantified the wavelength and intensity response of photothermal and photoelectronic effects in a plasmonic system, separately." This claim does <u>disservice</u> to my colleagues in the field who have <u>quantified</u> these effects, in some cases as a function of wavelength and intensity.*
We read all the five papers again very carefully. In fact, most of them were published in 2018, and one of them were published after the submission of our work (ACS Nano, 2018, 12, 8447–8455). Papers 1, 2 and 4 are all based on temperature control or detection. Paper 1 is using the extinction spectroscopy to compare the growth rates of Ag shell on Au cores in the dark at different temperatures with the one under plasmon excitation at different laser intensities. Paper 2 separates the reaction bed into the thermal and non-thermal effects by measuring the total reaction rate and the temperature gradient of the catalyst bed to extract the effective thermal reaction rate. They have to assume that no thermal effects occur in the non-thermal reaction part, which is inconsistent with the real situation. Paper 4 focuses on detecting the activation energies of plasmonic catalysts. This work requires the accurate measurement of the the surface temperature of plasmonic catalysts, which is a great challenge. In fact, the temperature measured in this paper is the environment temperature rather than the surface temperature. There is no attempt in discussing the effects of wavelength in all these three papers. On the other hand, Papers 3 and 5 are using SECM to quantify photothermal heating at plasmonic

nanoparticles, which is elegant. But, this method was not developed to determine whether the chemical reaction is induced by the excited carriers or heating. Limited by the principle of SECM, SECM can only detect the product diffuses to the probe, which will be influenced by the mass transport. Therefore, it is challenging for this method to quantify the wavelength and intensity responses of the photothermal and photoelectronic effects in the plasmonic system.
It should be pointed out that the continuous publication of papers in these high impact journal related to the topic also indicates the important and necessity of this work. However, we have to state that none of these papers were able to establish a **quantitative wavelength and intensity response of photothermal and photoelectronic effects**, because it is truly difficult to confidently separate the photothermal and photoelectronic effects and quantify their contribution on chemical reactions. Even in a very recent paper published during the reviewing process of our work, the authors tried to separate the hot carrier and thermal contributions in plasmonic photocatalysis (Science, 2018, 362, 69–72). Unfortunately, they are still not able to quantify wavelength and intensity response of photothermal and photoelectronic effects concurrently. Therefore, any efforts towards this goal will be highly appreciated by the field.

For clarity, we made the following revisions on page 1, line 22 in the manuscript:
"Although many efforts have been made, it is still a great challenge to confidently separate these two effects and quantify their relative contribution to chemical reactions."
and page 12, line 303 in the manuscript:
"we were able to disentangle the coupled photoelectronic and photothermal effects quantitatively, potentially guiding one's ability to optimize the design of plasmonic nanomaterials for specific applications,"

4) The authors themselves published a paper in 2015 called "Quantitative detection of photothermal and photoelectrocatalytic effects induced by SPR from Au@Pt nanoparticles". The current work is an increment over this published paper.

Response 3:
In this paper we mainly used surface-enhanced Raman scattering (SERS) to detect the photothermal and photoelectrocatalytic effects induced by SPR from Au@Pt NPs, and calculated the contribution of the ratio of the photothermal and photoelectrocatalysis effects by carrying out reaction at different temperature. The photothermal effect on the nanoparticle surface after illumination is detected by SERS. The photoelectrocatalytic effect generated from SPR is proved by SERS with a probe molecule of p-aminothiophenol (PATP). In the system, we have not been able to quantify the wavelength and intensity response of photothermal and photoelectronic effects in a plasmonic system, separately. Furthermore, we did observe the two-stage current response. However, we were not able to make any convincing assignment of the origin at that moment. In contrast, in the current work we for the first time are really able to systematically investigate into the current response and the influencing factors and distinguish their roles in plasmonic chemistry system.

5) The assignment of the slow and fast responses to photothermal and photocarrier effects do not have any technical or evidentiary basis. I raised this question and the authors have not

been able to answer it. When I read their response, I recognized that there is some confusion about this point, which continues to persist. The authors say “For the thermal effect, heat transfers to the surroundings on a longer timescale (nanoseconds). However, a longer time, usually tens of seconds, is needed to reach the thermal diffusion equilibrium with its local surroundings…” ***Likewise, the response to photocarrier processes can take a long time to reach steady-state. While e-ph relaxation times are on the order of 1 ps, the timescale to reach a steady-state in response to the photocarriers will depend on the timescale of electrochemical reactions and diffusion of charged intermediates. This timescale can be in the microseconds, milliseconds, or even seconds range. It simply depends on the timescale of reactions and diffusion just like the timescale for thermal steady-state to be reached in the medium depends on thermal conductivities and heat transfer coefficients***. Therefore, the interpretation “...photoelectrochemical method with a millisecond time resolution would be able to disentangle the charge carrier from photothermal effects.” is not evidence-based but guess-work-based and a bit naive. This argument is the central piece in their analysis, so the entire paper rests on shaky ground, in my opinion.

Response 4:

The main content of the question is bolded. The key point is: whether the slow response current is due to the electrochemical reactions and/or the diffusion of charged intermediates. This question is similar to Question 14 of Reviewer 2, which has been answered as follows in our previous response:

“We have also considered the reaction-diffusion process, but we still think it is not responsible for the slow response current for the following three reasons:

(1) Reaction-diffusion commonly exists in all photoelectrochemical systems. However, no slow response current (SRC) have been observed under similar conductions for the semiconductor photoelectrode.

(2) As mentioned by the referee, the slow diffusion of molecules/ions could yield a slow response of the photocurrent. Thus the time scale of the hydrogen evolution reaction at -0.6 V involving the proton diffusion should be faster than that of the oxygen reduction at 0 V. But the experimental result is just the opposite as shown in Figure 2b.

(3) The excellent fit of the experimental SRC to the derived thermal dynamics equations also supports the conclusion that the SRC is induced by the photothermal effect.”

Probably we still did not make the points sufficiently clear, we emphasize some key points as follows in a different way:

The Reviewer 1’s statement may be true only when the timescale of electrochemical reaction is in seconds in some very special system that can stabilize the generated charge, such as photosynthetic system. In 0.2 M sodium sulfate with a Pt foil counter electrode and a saturated calomel reference electrode, which is one of the most investigated photoelectrochemical system, only rapid response current (RRC) was observed in our study as well as in the literature. This response has been attributed to excited carriers in most of literatures including Ref.29-40. Reviewer 1 also emphasized the effect of the diffusion of the charged intermediate. We think the diffusion of the charged intermediate is not responsible for the slow response current for the following three reasons: (1) The charge transfer from the electrode to the reactant happens on the electrode surface, thus the diffusion of the charged intermediates from the electrode

surface to electrolyte will decrease the current. But in our system, the SRC is increased. (2) The lifetime of charged intermediates is usually in μs or ms, which does not match the time scale of the diffusion process. Thus, it is very unlikely that the diffusion of the charged intermediates can lead to the slow response of several seconds. (3) If the response is due to the diffusion of the charged intermediates or electrochemical reactions, we should be able to observe the slow response current (SRC) in all photoelectrochemical systems. However, no SRC have ever been observed under similar conductions for the semiconductor photoelectrode. In addition, the following three evidences are strong supports to the above assignment that the SRC is a result of photothermal effects. First, the SRC strongly depends on the electrochemical reaction. Second, the experimental SRC as a function of time excellently follows the photothermal dynamics. Third, the experimental SRC (read from the photothermal current) linearly depends on the intensity of the incident light (the SRC in Figure R2), which agrees well with the theoretical thermal dynamics:

$$I = A_0(Ae^{-\frac{ak}{Cl}t} + \frac{Pl}{ak} + T_0) + D$$

On the other hand, RRC shows a totally different dependence.

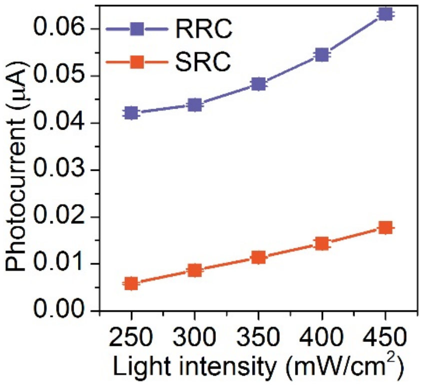

Figure R2: the RRC and SRC as a function of the incident light intensity

For clarity, we made the following revisions on page 4, line 98 in the manuscript:

"As has been well observed and investigated in semiconductor electrodes or plasmonic metal-semiconductor complexes,[12,13,39,40] the photocurrent induced by energetic charge carriers responds immediately upon illumination, and reaches maximum usually in less than several ms (Figure 1e2)."

6) I had asked "In Fig. 4b, the red data points describe the photothermally induced photocurrent as a function of the excitation wavelength. Why does this plot not follow the absorption spectrum? Photothermal temperature rise would be expected to track the absorption cross-section." The response was "The absorption spectrum shown in manuscript is in fact the extinction spectrum, which includes absorption and scattering. Photothermal effect is mainly dominated by the absorption and may not necessarily follow the line-shape of the extinction spectrum." I have experience measuring such spectra. The line-shape and peak maximum location (550 nm) of the extinction spectrum is similar to what is expected for the absorption spectrum in this case. It would have been quite simple for the authors to measure the pure absorption spectrum by diffuse reflectance and they would find that it is quite similar in shape to the extinction spectrum.

But the authors did not measure the absorption spectrum. So, really the authors' response is, in my opinion, equivalent to sweeping the issue under the rug. So, once again, let me state the problem: the photothermal temperature rise does not track the spectrum (extinction or absorption), which indicates that the authors are not measuring the photothermal temperature rise. I hope the authors take this issue seriously.

Response 5:

This question is mainly on why the photothermal current does not track the spectrum. The reviewer's opinion will be valid only when all the absorbed light is converted into heat. But, this is almost impossible for a plasmonic chemistry system, because if the energetic excited carriers participate in the chemical reaction, they will not further thermalize. For the incident light with wavelengthes shorter than 500 nm (corresponding to the d to s band interband transition), excited electrons are generated in the vicinity of the Fermi level (i.e. with low energy) which quickly thermalize. So the photothermal effect is more obvious (Refs. 11, 12, 13, 23). So the photothermal effect is more obvious (Ref. 11, 12, 13, 23). In contrast, when the absorbed light is resonant with the SPs (intraband region), the generated excited carriers participated the chemical reaction. (Figure R5b, the blue dot)

Following the reviewer's suggestion, we also tried to measure the "absorption spectrum" by diffuse reflectance, as shown in Figure R3a. We only observed a strong absorption on 520 nm. But we did not observe the absorption in the interband transition region at wavelengths shorter than 500 nm. In addition, we also obtained an abnormally strong absorption after 650 nm.

According to our understanding, the diffuse spectroscopy requires uniform scattered light. However, our periodic substrate has a strong directional reflection, which does meet the basic requirement of diffuse reflectance measurement. Therefore, in the literature, reflectance spectrum was used to characterize similar systems instead of diffuse reflectance spectrum. (Ref 41-43; Nat. Mater. 2016, 15, 621-627; Nat. Commun. 2018, 9, 2160; Science 2018, 360, 1105-1109)

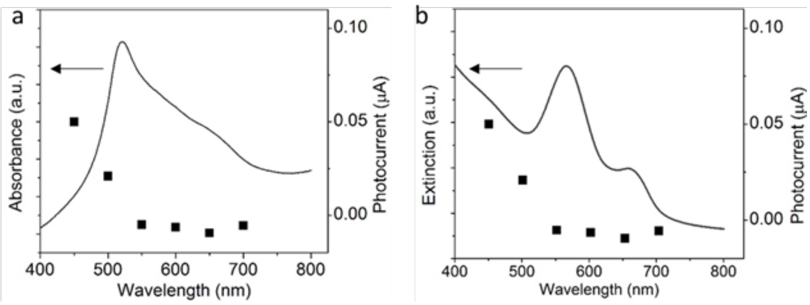

Figure R3. (a) The absorption spectrum measured by diffuse reflectance. (b) The extinction spectrum detected by the specular reflection model

We have made a number of attempts to measure the temperature of the system. But the photothermal effect of a plasmonic nanostructure is confined to nanometer range and shows gradient. Thus, it is very challenging to detect the local temperature (surface or interface temperature) and the gradient of the thermal field (Ref 11). In our photoelectrochemical experiments using a chopped incident light, there is no obvious temperature increase in the electrolyte under each cycle of "on and off" the light. In fact, recently we developed a method

to measure the surface temperature of plasmonic nanoparticles with strong SERS enhancement using an isocyanide molecule by measuring the frequency shift of SERS peak (J. Am. Chem. Soc. 2018, 140, 13680−13686). However, the plasmonic electrode used in this study did not show sufficient SERS enhancement to allow us to use the above method to measure the temperature.

For clarity, we made the following revisions on page 5, line 111 in the manuscript:
"the UV-Vis distinction spectrum detected by the specular reflection mode in air and in 0.2 M sodium sulfate solution"
and page 10, line 262:
"LSPR part"
and page 10, line 267：
"Whereas, under the illumination with light of a longer wavelength (LSPR part), the energetic excited carriers of SPs participate in the chemical reaction, they will not further thermalize (Figure 4b)."

7) One of the author's last response to me states "The excited carriers generated under longer wavelength usually have a lower energy, which decreases the probability to induce chemical reactions." This is demonstrably incorrect. Several groups have shown chemical reactions induced by excitation at longer wavelengths (650 nm, for instance which matches with the longer-wavelength resonance in the authors' work): e.g.

https://pubs.acs.org/doi/full/10.1021/acscentsci.7b00122

I feel that the authors are choosing to ignore published evidence to justify ignoring an important anomaly in their results.

Response 6:
The Reviewer 1' comment is "*Several groups have shown chemical reactions induced by excitation at longer wavelengths*". But, in our periodic plasmonic substrate, the plasmon resonance mode at longer-wavelengths in our work is attributed to surface plasmon polariton (SPP) of the periodic array rather than the LSPR. Up to now, there is no any report on the possibility of using SPP to assist plasmon chemistry.
Additionally, concerning the possibility of reaction induced by the longer wavelength, in fact in our previous response, we only stated "decreases the probability to induce chemical reactions" and we did not exclude the possibility. It should be pointed out that the excited carriers generated under a longer wavelength usually have a lower energy, which decreases the probability to induce chemical reactions. This is also correct in the papers suggested by the Reviewer 1 (Figure R4) as well as in other photocatalytic systems, the reaction probability of chemical reactions induced by excitation at longer wavelengths decreases greatly.

[redacted]

Figure R4.

For clarity, we made the following revisions on page 10, line 263 in the manuscript:
"We did not find particular enhancement in photocurrent for the 650 nm peak, attributed to the surface plasmon polariton (SPP) of the periodic array."

8) MINOR POINT: Fig. 4b is still incorrect. In a rigorous electron transfer theory depiction, the initial potential energy surface is that of all reagents (including solvent) prior to electron transfer. The final potential energy surface is that of all reagents (including solvent) post-electron transfer. The authors label the initial potential energy surface as "reactant" and the final potential energy surface as "product", which is a misunderstood version of potential energy surfaces mean in electron transfer. Given the other weakness of the paper, this point is relatively minor, especially because this mistake is common, but as a physical chemist I felt it obligatory to point out the issue. The x-axis is the reaction (configurational) co-ordinate, which the authors choose not to label.

Response 7:
We thank the referee for the comment. It's true that "the initial potential energy surface is that of all reagents (including solvent) prior to electron transfer". However, in most cases the reactant makes the main contribution and the solvent effect can be neglected. Thus, in the common practice in the field, the schematic potential energy surface usually does not show the solvent to simplify the understanding. This is the case for some leading groups in the field, including Halas (Nano Lett. 2013, 13, 240−247) and Linic (Nature Mater. 2015, 14, 567-576) groups. We prefer to use such a widely used and simplified expression for an easy understanding by the readers.

For clarify, we made the following revisions on page 11, line 285 in the manuscript:
"The schematic potential energy surface was simplified by using "Reactant" and "Product"."
and we further revise the overlapped curve as dash curve to avoid any possible misunderstanding (see Figure c in the Figure R5). A similar description has also been commonly used in literatures (For example, Chem. Rev. 1991, 91, 767-792; Nano Lett. 2013, 13, 240−247).

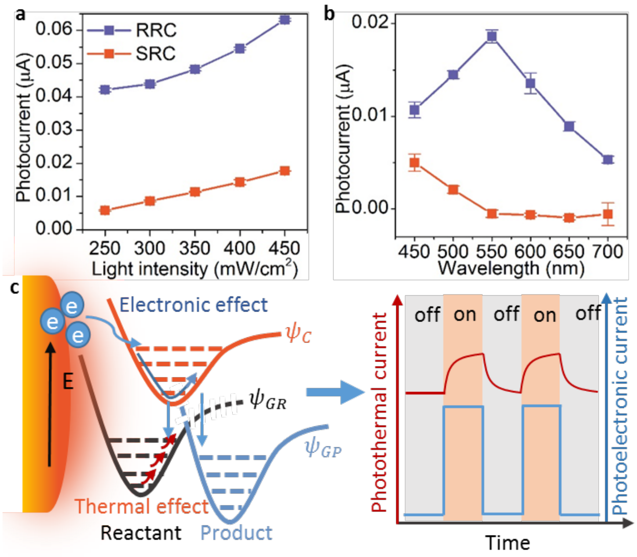

Figure R5.

Reviewer #2 (Remarks to the Author):

The authors properly answered my concerns.

Thank you for your comments.

Reviewer #3 (Remarks to the Author):

The authors have addressed all comments in a satisfactory way and the manuscript has improved substantially. I recommend publication as it is now.

Thank you for your comments.

Reviewers' comments:

Reviewer #1 (Remarks to the Author):

Once again I thank the authors for their responses to my questions. Unfortunately, my view about the paper does not change, primarily because I do not find the paper to be improved or the deficiencies to be addressed.

The authors have not been able to verify the putated photothermal contribution by a temperature measurement. Not only the local but the global medium temperature is also expected to rise under light excitation due to faster buildup of heat in the medium as compared to heat loss from the reaction cell. The authors indicate that there is 'no obvious temperature rise'. While this is bothersome, this limitation in the study ought to be described in the manuscript. This is information to important to be left out of the manuscript.

I had rasised a question about the lack of a peak in photoactivity at 650 nm. The diffuse reflectance spectrum described in the paper does show significant response at 650 nm, yet no photothermal contribution is measured at 650 nm. This is also quite bothersome in my opinion. Regardless of the assignment of a feature in an absorption spectrum, high absorption ought to be directly correlated with photothermal heating. The authors point out that the high absorption at 650 nm may simply be artefact due to directional reflection. If the authors are sure about this explanation, this point ought to be described in the manuscript.

I also noted from Figure R3 that the photothermal contribution rises above 650 nm. This effect is inconsistent with the explanations in the paper.

The authors acknowledge that plasmonic metal electrodes have been designed and studied, but the novel aspect of the current study is that these electrodes were not supported on a semiconductor. If this is the case, a major discussion point ought to be how the results and findings are different in the current study as compared to studies of plasmonic nanoparticles supported on the semiconductor. I did not find any such discussion added to the manuscript. The long response of the author is not accompanied by a proportionate revision in the manuscript.

The previous studies on separation of thermal and non-thermal effects in plasmon enhanced catalysis have been critiqued in the authors' response and limitations of these studies have been pointed out. Such a discussion ought to be included in the manuscript to emphasize the novelty of the authors' current study. Once again, the long response of the author is not accompanied by a proportionate revision in the manuscript.

The authors also say that unlike the current work "There is no attempt in discussing the effects of wavelength in all these three papers." They refer to Papers 1, 2, and 4. Actually , paper 4 appears to compare interband and plasmonic excitation wavelengths. Paper 2 compares UV and blue excitation. I am not able to take the response of the authors at its face value.

The authors state as a new point that "the plasmonic electrode can function as not only a photocathode (a source of excited electrons) but also a photoanode (delivering excited holes for an oxidation reaction), which has never been observed in semiconductors and plasmon-semiconductor complexes." This is not accurate. Semiconductor photoanodes are common in water splitting, e.g., https://pubs.acs.org/doi/abs/10.1021/jp9041974. Even in the realm of plasmonic metals, this aspect is not new. For example, Moskovits and coworkers reported in 2013 "...autonomous solar water-splitting device based on a gold nanorod array in which essentially all charge carriers involved in the oxidation and reduction steps arise from the hot electrons resulting from the excitation of surface plasmons in the nanostructured gold." Once again, I am not able to take the response of the authors at its face value.

On a separate note, I also feel that the responses and revisions lack care. For instance, the editorial policy checklist filled out by the authors checks the box "Code availability statement" but I did not find such a statement in the manuscript. Likewise I did not see the use of dot plots, individual data points, box and whisker plots, definition of error bars, or disclosure of sample size n. Despite the lack of this information, the authors chose to check the "Yes" boxes in the policy checklist.

Reviewers' comments:

Reviewer #1 (Remarks to the Author):

Once again I thank the authors for their responses to my questions. Unfortunately, my view about the paper does not change, primarily because I do not find the paper to be improved or the deficiencies to be addressed.

The authors have not been able to verify the putated photothermal contribution by a temperature measurement. Not only the local but the global medium temperature is also expected to rise under light excitation due to faster buildup of heat in the medium as compared to heat loss from the reaction cell. The authors indicate that there is 'no obvious temperature rise'. While this is bothersome, this limitation in the study ought to be described in the manuscript. This is information to important to be left out of the manuscript.
Response 1:
Suggested by the reviewer, we detected the global medium temperature under the chopped incident light (300 mW/cm^2, which is of the order of the power density of sunlight and about several orders of magnitude smaller than that of focused laser.) in our system. It is obvious that the temperature increase is only about 0.03 K under our experimental condition, which is very small. Thus we conclude that there is 'no obvious temperature rise'.

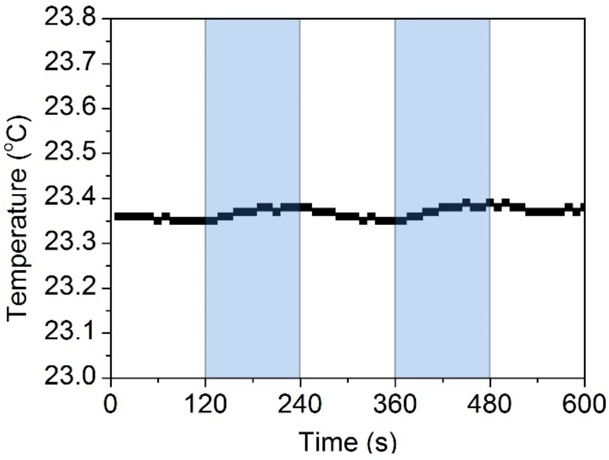

Figure R1: Change of the medium temperature with the chopped incident light.

Now we added it in the manuscript on page 7, line 111 as well in the SI:
"Additionally, the temperature increase (~ 0.03 K, Figure S12) in the solution is negligible under our experimental condition."

I had raised a question about the lack of a peak in photoactivity at 650 nm. The diffuse reflectance spectrum described in the paper does show significant response at 650 nm, yet no photothermal contribution is measured at 650 nm. This is also quite bothersome in my opinion. Regardless of the assignment of a feature in an absorption spectrum, high absorption ought to be directly correlated with photothermal heating. The authors point out that the high absorption at 650 nm may simply be artefact due to directional reflection. If the authors are sure about this explanation, this point ought to be described in the manuscript.

Response 2:

The reviewer's comment is mainly on why the photothermal current (SRC) does not track the diffuse reflectance spectrum for the shoulder band at 650 nm. As we have described in the manuscript, the 650 nm band is mainly contributed by the surface plasmon polariton (SPP) of the periodic array. In our previous response to the reviewer, we have pointed out that "*our periodic substrate has a strong directional reflection, which does not meet the basic requirement of diffuse reflectance measurement*." Therefore, even we could obtain a side band for the wavelength at around 650 nm in the diffuse reflectance spectrum, it did not correctly reflect the absorption feature of the substrate. Additionally, the reviewer's opinion "*high absorption ought to be directly correlated with photothermal heating*" will be valid only when all the absorbed photon energy is converted into heat during the relaxation of excited carrier (*Nature Nanotech*. 2015, **10**, 25; *Annu. Rev. Phys. Chem*. 2017, **68**, 379; *Nat. Rev. Chem*. 2018, **2**, 216). However, this becomes complicated for a plasmonic chemistry system, where the energetic excited carriers participate in the chemical reaction. These carriers will not further thermalize.

If the reviewer's comment "*I had raised a question about the lack of a peak in photoactivity at 650 nm.*" is concerned about the photoelectronic effect, it addressed the RRC response curve. In fact, we did see a significant RRC response and even a small slope change at around 650 nm. The following three more reasons may lead to the small deviation of RRC curve to the extinction spectrum: (1) The excited carriers generated under a longer wavelength usually have a lower energy, which decreases the probability to induce chemical reactions. (2) The plasmonic photochemistry process involves the complicated interplay of surface plasmons, excited carriers, heat, and chemical reactions (*Nature Chem*. 2011, **3**, 467; *J. Am. Chem. Soc*. 2011, **133**, 5202; *J. Am. Chem. Soc*. 2014, **136**, 64; *Nature Photon*. 2016, **10**, 473). (3) The effect of SPP on chemical reaction is still not conclusive.

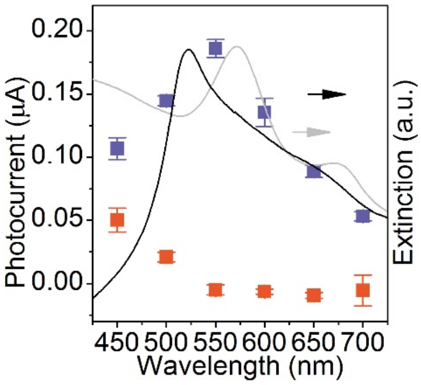

Figure R2: The RRC and SRC as a function of the incident light wavelength. The black one is the absorption spectrum measured by diffuse reflectance. The gray one is the extinction spectrum detected by the specular reflection model.

For clarify, we added a description for the diffuse spectroscopy and reflectance spectroscopy in page 2 in the SI.

"The reflection model is chosen because that our periodic substrate has a strong directional reflection, which does not meet the basic requirement of diffuse reflectance measurement."

And we revised the Figure 4b in the manuscript, and added the description in page 11, line 265:

“We did see a small deviation of RRC curve to the extinction spectrum, which is understandable, as the PMCR system is a complex reaction system including surface plasmons, excited carriers, local heating and chemical reactions.[7,37,46] Two more facts may further contribute to the deviation: one is the energy distribution of the excited carriers generated under different wavelength which can influence the probability to induce chemical reactions and the other is the effects of SPP on the chemical reaction.”

We also added a discussion for the photothermal and photoelectronic current in the manuscript on page 11, line 273:

“the energetic excited carriers of SPs participate in the chemical reaction, they will not further thermalize (Figure 4b). This is a unique feature of excited carriers and photothermal effect in the PMCR system.”

I also noted from Figure R3 that the photothermal contribution rises above 650 nm. This effect is inconsistent with the explanations in the paper.

Response 3:

The photothermal current shown in Figure R3 of previous response is the average value of SRC in Figure R2 (Figure 4b in manuscript). All values of photothermal current are negligible (~ 5 nA) when the incident light wavelength is longer than 550 nm, which is about an order of magnitude smaller than that under 450 nm illumination. When the wavelength is longer than 650 nm, the photothermal current is also very small, and the slight increase is mainly from the measurement error of current. Thus we can't conclude that the photothermal contribution rises above 650 nm.

The authors acknowledge that plasmonic metal electrodes have been designed and studied, but the novel aspect of the current study is that these electrodes were not supported on a semiconductor. If this is the case, a major discussion point ought to be how the results and findings are different in the current study as compared to studies of plasmonic nanoparticles supported on the semiconductor. I did not find any such discussion added to the manuscript. The long response of the author is not accompanied by a proportionate revision in the manuscript.

Response 4:

We thank the reviewer for the helpful comment. We added the comparison in the manuscript on page 3, line 63:

“For example, the contact between the plasmonic nanostructure and semiconductors can greatly influence the charge transfer process.[23]”

And on page 6, line 145:

“For plasmonic metal-semiconductor electrode, the direction and efficiency of the charge transfer process are highly related to the band structure of the semiconductor and the Fermi level of the plasmonic nanostructure. Moreover, the interface between them can also greatly influence the charge transfer direction and efficiency, which is yet to be more extensively investigated and sophisticatedly controlled.”

The previous studies on separation of thermal and non-thermal effects in plasmon enhanced catalysis have been critiqued in the authors' response and limitations of these studies have been pointed out. Such a discussion ought to be included in the manuscript to emphasize the novelty of the authors' current study. Once again, the long response of the author is not accompanied by a proportionate revision in the manuscript.

Response 5:

We thank the reviewer for the helpful comment. We added this comparison in the manuscript on page 2, line 48:

"Recently, some elegant attempts have been made to address the challenge of distinguishing excited carriers from thermal effects.[14,25-28] One typical strategy is based on temperature control and detection. For example, it was found that the incident light can reduce the thermal activation barrier for ammonia decomposition on a plasmonic photocatalyst.[28] But this strategy requires the accurate measurement of the surface/local temperature of plasmonic catalysts, which is still a great challenge. Another example is using scanning electrochemical microscope (SECM), which has been successfully used to investigate the roles of photoinduced reactions at the substrate and enhanced mass transport to the SECM tip due to the local heating.[26] It is still difficult for SECM to determine whether the chemical reaction on surface is induced by the excited carriers or heating, because SECM can only detect the product diffusing to the probe. Investigating these two effects separately and quantitatively is highly desirable, which enables one to extract the key factors that influence the plasmon-mediated processes."

The authors also say that unlike the current work "There is no attempt in discussing the effects of wavelength in all these three papers." They refer to Papers 1, 2, and 4. Actually, paper 4 appears to compare interband and plasmonic excitation wavelengths. Paper 2 compares UV and blue excitation. I am not able to take the response of the authors at its face value.

Response 6:

Sorry for the misleading statement of the last sentence due to the incomplete sentence. Indeed, what we exactly meant is: "There is no attempt in discussing the effects of wavelength on the photothermal and photoelectronic effects separately in all these three papers" in response to the question "*quantified the wavelength and intensity response of photothermal and photoelectronic effects in a plasmonic system*". In Paper 2, the authors gave the reaction rate as a function a light intensity under UV and blue excitation, but they did not separate the photothermal and photoelectronic effects (Figure R3a). In paper 4, the authors tried to compare the apparent activation energy of the catalyst at two different excitation wavelengths. Again they didn't discuss the effects of wavelength on the photothermal and photoelectronic effects separately (Figure R3b).

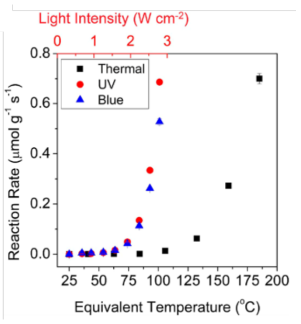

[redacted]

Figure R3: a, Overall low temperature reaction rates for dark heating conditions (black squares) and under UV (red circles) and blue illumination (blue triangles) without additional heating (paper 2). b, Increase in light excitation power causes a reduction in the apparent activation enthalpy (paper 4). Part a is reprinted with permission from *Nano Lett.* **18**, 1714-1723 (2018). Copyright (2018) American Chemical Society.

The authors state as a new point that "the plasmonic electrode can function as not only a photocathode (a source of excited electrons) but also a photoanode (delivering excited holes for an oxidation reaction), which has never been observed in semiconductors and plasmon-semiconductor complexes." This is not accurate. Semiconductor photoanodes are common in water splitting, e.g., https://pubs.acs.org/doi/abs/10.1021/jp9041974. Even in the realm of plasmonic metals, this aspect is not new. For example, Moskovits and coworkers reported in 2013 "...autonomous solar water-splitting device based on a gold nanorod array in which essentially all charge carriers involved in the oxidation and reduction steps arise from the hot electrons resulting from the excitation of surface plasmons in the nanostructured gold." Once again, I am not able to take the response of the authors at its face value.

Response 7:

We thank the reviewer for the thoughtful comment and we respectfully disagree with the reviewer that our statement "*the plasmonic electrode can function as not only a photocathode (a source of excited electrons) but also a photoanode (delivering excited holes for an oxidation reaction), which has never been observed in semiconductors and plasmon-semiconductor complexes.*" is inaccurate. Indeed, this work is the first one pointing out that plasmonic metals can act as both photoanode and photocathode. We detail our reasons as follows:

1. In the JPC paper suggested by the reviewer (Figure R4), TiO_2 only acted as photoanode to accelerate the oxidation reaction and the reduction took place on the platinum counter electrode. This is similar to the first TiO_2 photoelectrohemistry paper of Fujishima (Nature, 1972, 238, 37–38), in which the n-type TiO_2 acted as photoanode for the water oxidation and Pt was the cathode for hydrogen evolution. However, n-type TiO_2 cannot be used as a photocathode, as there was no obvious photocurrent response at the cathodic polarization under illumination, as shown in Figure R5.

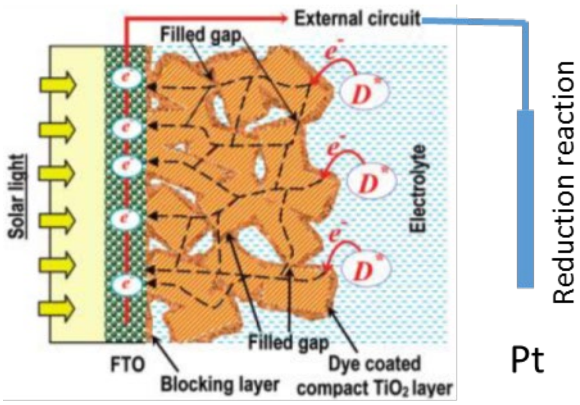

Figure R4: Schematic diagrams of electron pathways of the sol modified TiO_2 electrode acting as a photoanode. Reprinted with permission from *J. Phys. Chem. C*, **113**, 16277-16282 (2009). Copyright (2009) American Chemical Society.

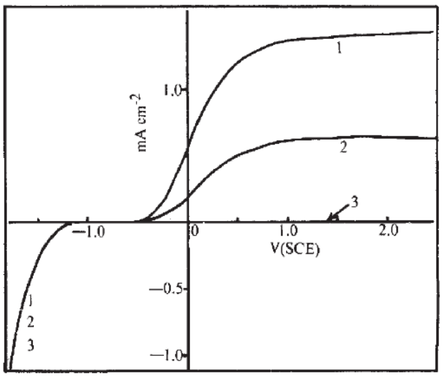

Figure R5: Current-voltage curves for TiO_2 under different conditions. 1 and 2 are under illumination. 3 is under the dark condition. Reprinted with permission from *Nature*, **238**, 37-38 (1972). Copyright (1972) Springer Nature Limited.

2. For the paper we published in 2013, we reported an autonomous photosynthetic device in which all charge carriers derive from surface plasmons. However, as seen from Figure R6b, the Au complex electrode (containing Au nanorodes, TiO_2 and Pt nanoparticles) was only used as a photocathode. We didn't explore the possibility of using it as a photoanode.

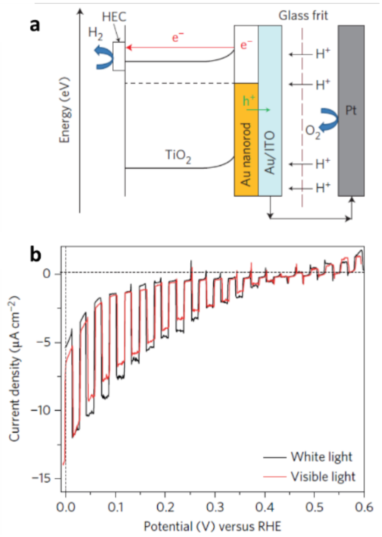

Figure R6: Schematic of the Au complex electrode acting as a plasmonic photocathode. Reprinted with permission from *Nat. Nanotech.* **8**, 247-251 (2013). Copyright (2013) Springer Nature Limited.

3. On contrast, in this work, we found the enhanced photocurrent of the plasmonic electrode can be both anodic and cathodic, indicating the plasmonic electrode can function as not only a photocathode but also a photoanode. Such a property has never been observed before and is different from semiconductors or plasmonic-semiconductor complexes.

On a separate note, I also feel that the responses and revisions lack care. For instance, the editorial policy checklist filled out by the authors checks the box "Code availability statement" but I did not find such a statement in the manuscript. Likewise I did not see the use of dot plots, individual data points, box and whisker plots, definition of error bars, or disclosure of sample size n. Despite the lack of this information, the authors chose to check the "Yes" boxes in the policy checklist.

Response 8:

We thank the reviewer for the comment. In this work, we didn’t use custom code or mathematical algorithm. We filled out the box of **code availability statement** (For all studies using custom code or mathematical algorithm that is deemed central to the conclusions, the Methods section must include a statement under the heading "Code availability" describing how readers can access the code, including any access restrictions.). And box-plot elements in the manuscript are defined, except the error bar. The error bars in the manuscript represent the relative deviation.

For clarify, we added it to the manuscript:

“The error bars in the manuscript represent the relative deviation.”
